# Supplementary material for: Design, Synthesis, and Mechanistic Study of 2-Pyridone-Bearing Phenylalanine Derivatives as Novel HIV Capsid Modulators
Source: Molecules. 2022 Nov 7;27(21):7640. doi: 10.3390/molecules27217640 (PMC9658817; doi:10.3390/molecules27217640)

Design, Synthesis, and Mechanistic Study of 2-Pyridone-bearing Phenylalanine Derivatives as Novel HIV Capsid Modulators

Xujie Zhang[a,†], Lin Sun[a,b,†], Shujing Xu[a], Xiaoyu Shao[a], Ziyi Li[a], Dang Ding[a], Xiangyi Jiang[a], Shujie Zhao[a], Simon Cocklin[c], Erik De Clercq[d], Christophe Pannecouque[d,*], Alexej Dick[e,*], Xinyong Liu[a,*], Peng Zhan[a,*]

[a]*Department of Medicinal Chemistry, Key Laboratory of Chemical Biology (Ministry of Education), School of Pharmaceutical Sciences, Shandong University, 44 West Culture Road, 250012 Jinan, Shandong, China.*

[b]*Department of Pharmacy, Qilu Hospital of Shandong University, 107 West Culture Road, Jinan 250012, Shandong, China.*

[c]*Specifica, Inc., 1607 Alcaldesa Street, Santa Fe, NM, 87501, USA*

[d]*Rega Institute for Medical Research, Laboratory of Virology and Chemotherapy, K.U. Leuven, Herestraat 49 Postbus 1043 (09.A097), B-3000, Leuven, Belgium*

[e]*Department of Biochemistry & Molecular Biology, Drexel University College of Medicine, Philadelphia, Pennsylvania, PA 19102, USA.*

*Corresponding authors. E-mail address: zhanpeng1982@sdu.edu.cn (Zhan P.); xinyongl@sdu.edu.cn (Liu X.Y.); ad3474@drexel.edu (Dick A.); christophe.pannecouque@kuleuven.be (Pannecouque C.).

†These authors contributed equally to this work as first authors.

1 MS, ^{1}H-NMR and ^{13}C-NMR Spectra for Representative Target Compounds

1.1 MS, ^{1}H-NMR and ^{13}C-NMR Spectra for FTC-2

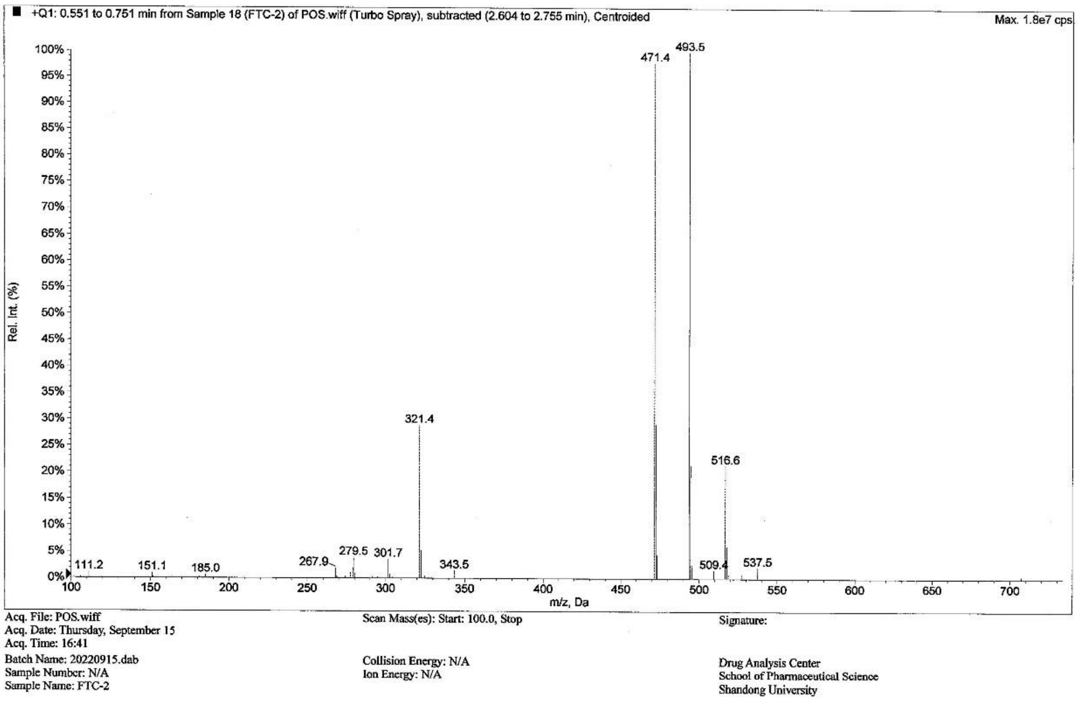

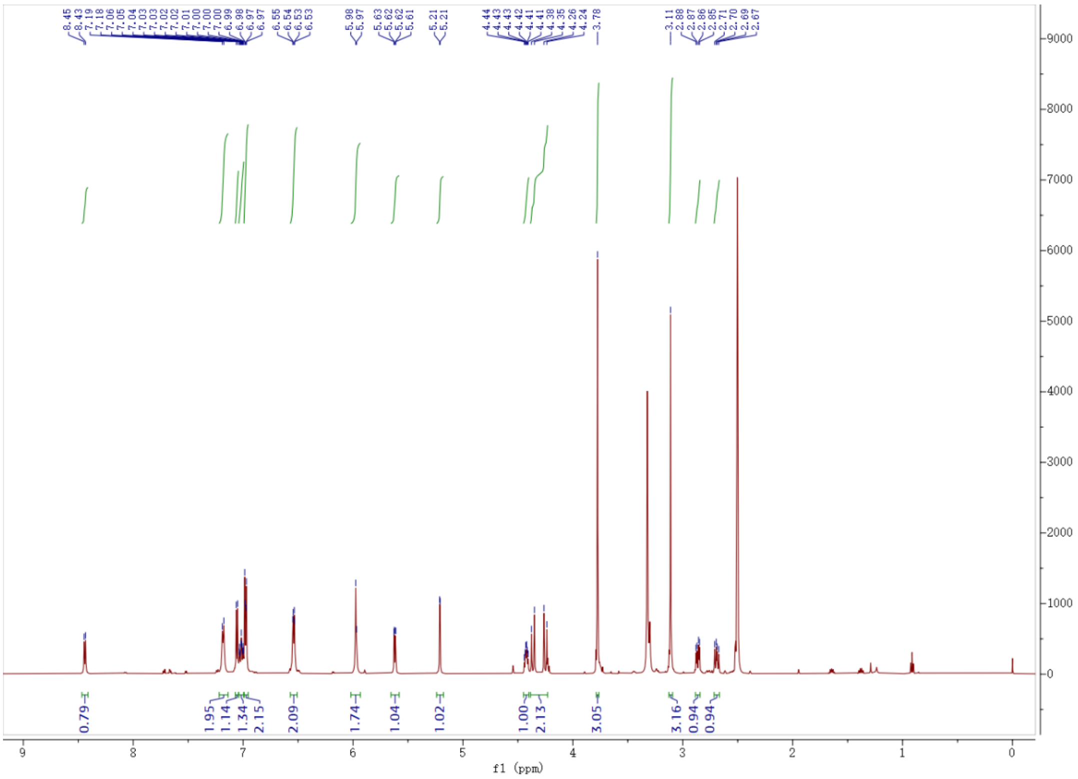

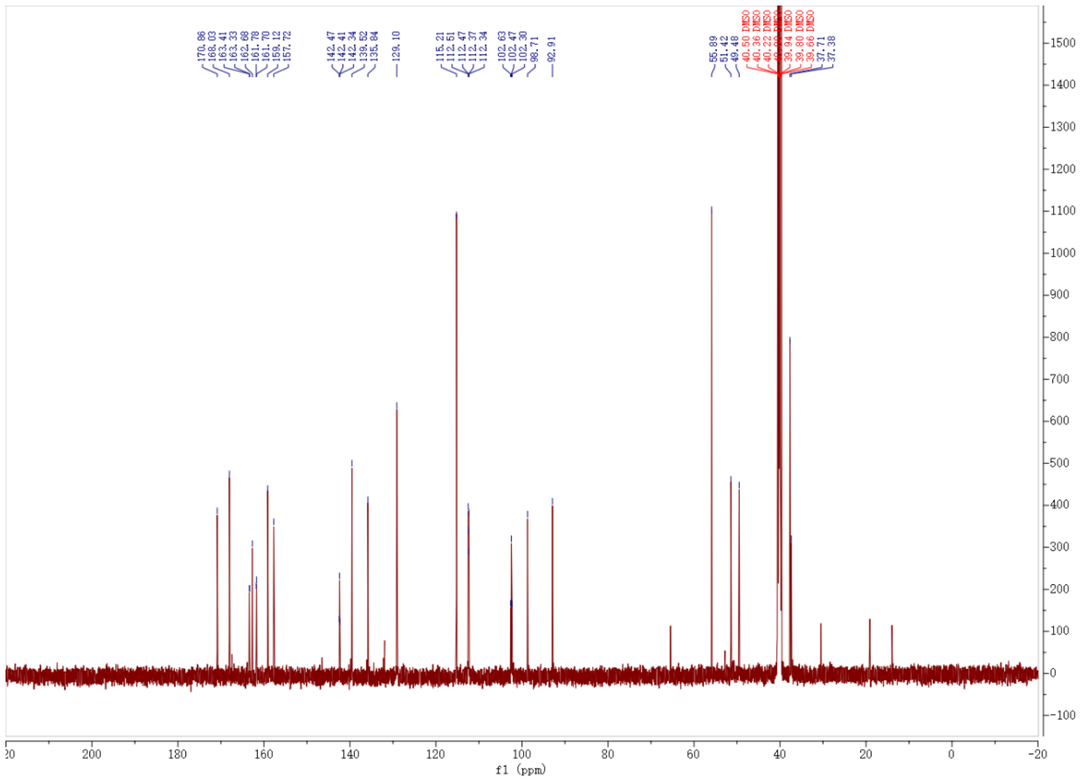

170.86
168.03
163.41
163.33
162.88
161.78
161.70
159.12
157.72
142.47
142.41
142.34
139.52
135.84
129.10
115.21
112.51
112.47
112.37
112.34
102.63
102.47
102.30
98.71
92.91
55.89
51.42
49.48
40.50 DMSO
40.36 DMSO
40.22 DMSO
39.94 DMSO
39.80 DMSO
39.66 DMSO
37.71
37.38
f1 (ppm)

1.2 MS, ^{1}H-NMR and ^{13}C-NMR Spectra for TC-2

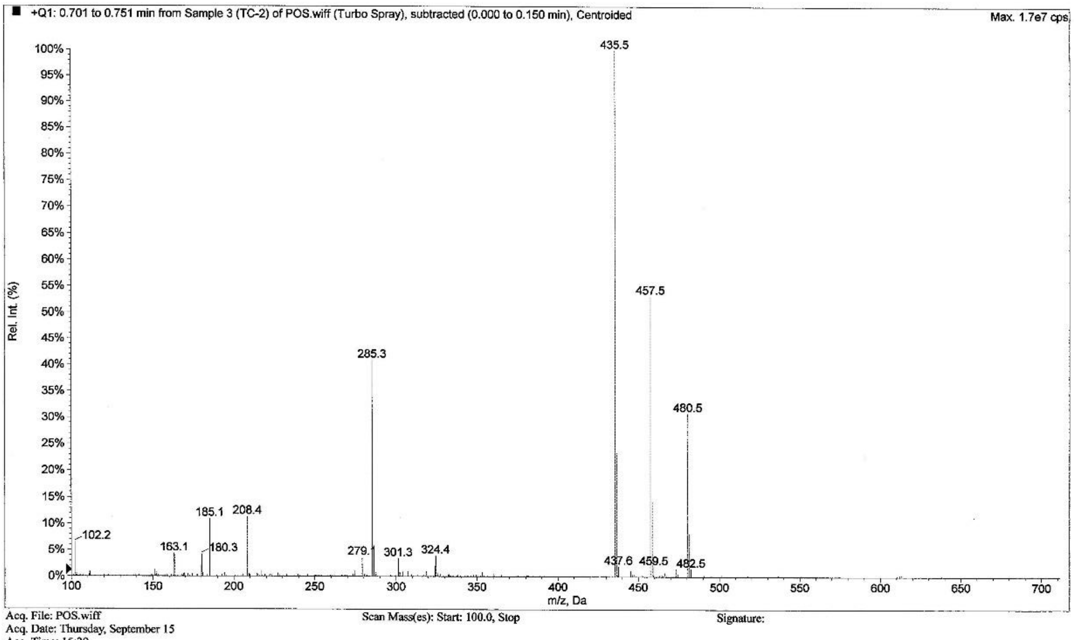

Acq. File: POS.wiff
Acq. Date: Thursday, September 15
Acq. Time: 15:39
Batch Name: 20220915.dab
Sample Number: N/A
Sample Name: TC-2

Scan Mass(es): Start: 100.0, Stop

Collision Energy: N/A
Ion Energy: N/A

Signature:

Drug Analysis Center
School of Pharmaceutical Science
Shandong University

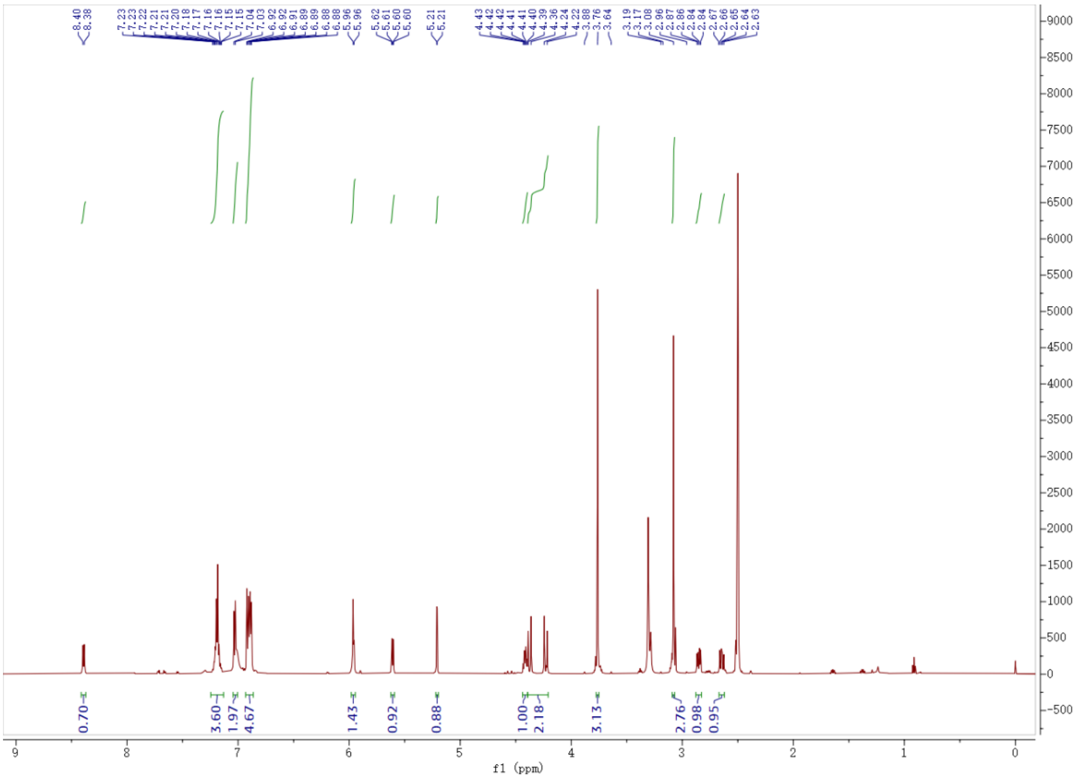

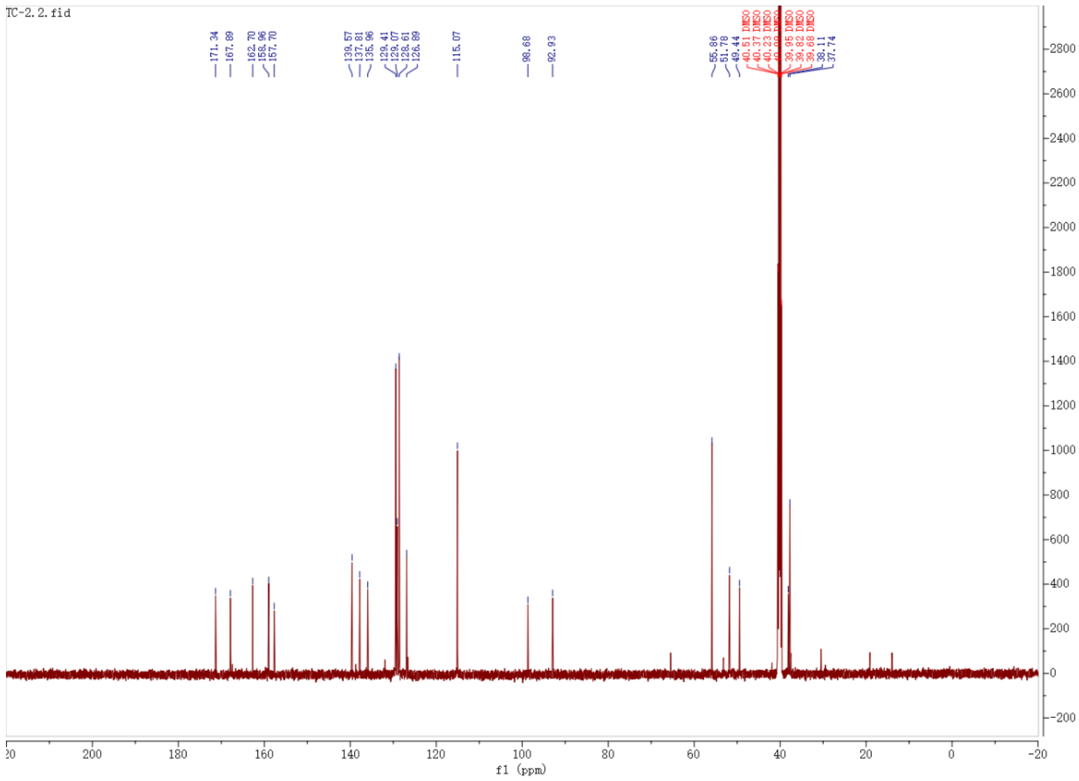
TC-2.2.fid
171.34
167.89
162.70
158.96
157.70
139.57
137.81
135.96
129.41
129.07
128.61
126.89
115.07
98.68
92.93
55.86
51.78
49.44
40.51 DMSO
40.37 DMSO
40.23 DMSO
39.95 DMSO
39.82 DMSO
39.68 DMSO
38.11
37.74
f1 (ppm)

1.3 MS, ^{1}H-NMR and ^{13}C-NMR Spectra for TD-1a

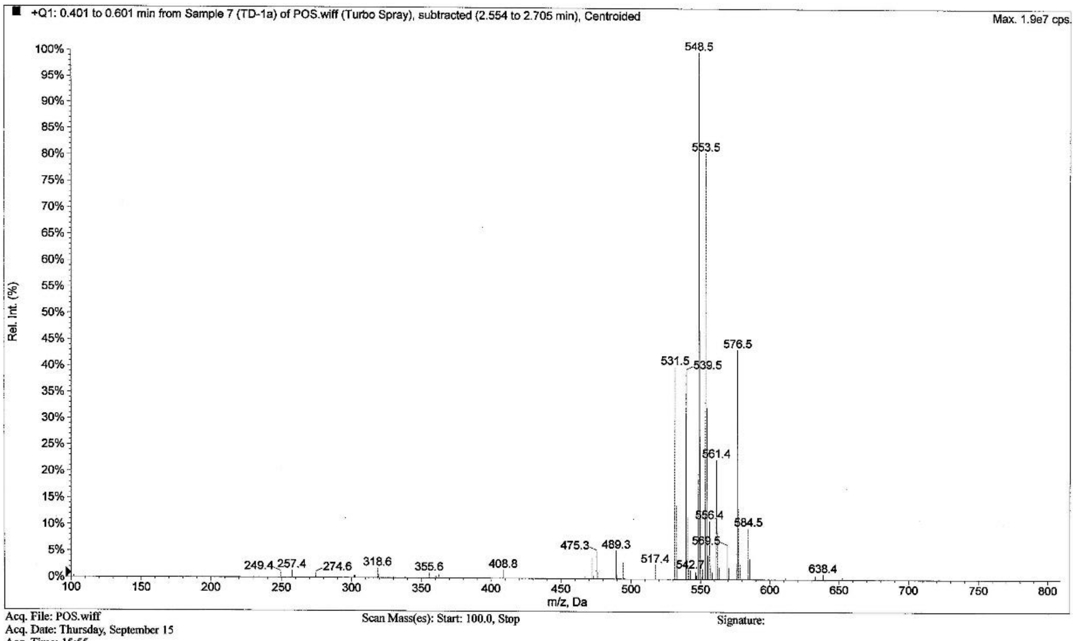

Acq. File: POS.wiff
Acq. Date: Thursday, September 15
Acq. Time: 15:55
Batch Name: 20220915.dab
Sample Number: N/A
Sample Name: TD-1a

Scan Mass(es): Start: 100.0, Stop

Collision Energy: N/A
Ion Energy: N/A

Signature:

Drug Analysis Center
School of Pharmaceutical Science
Shandong University

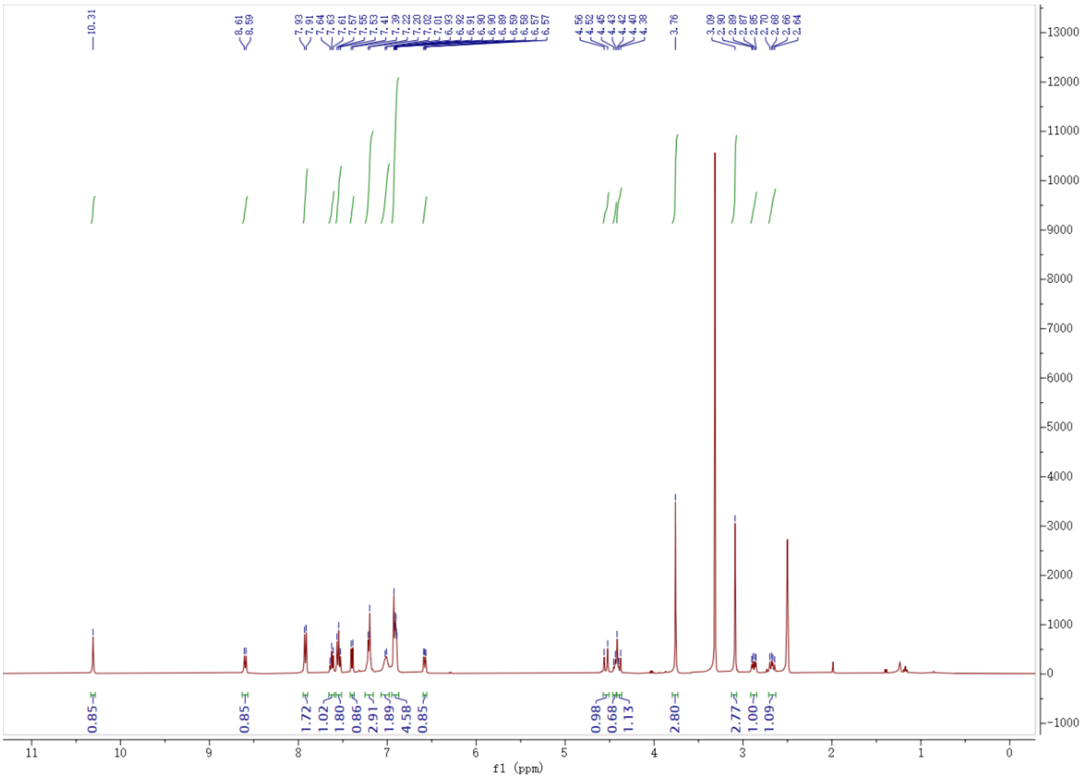

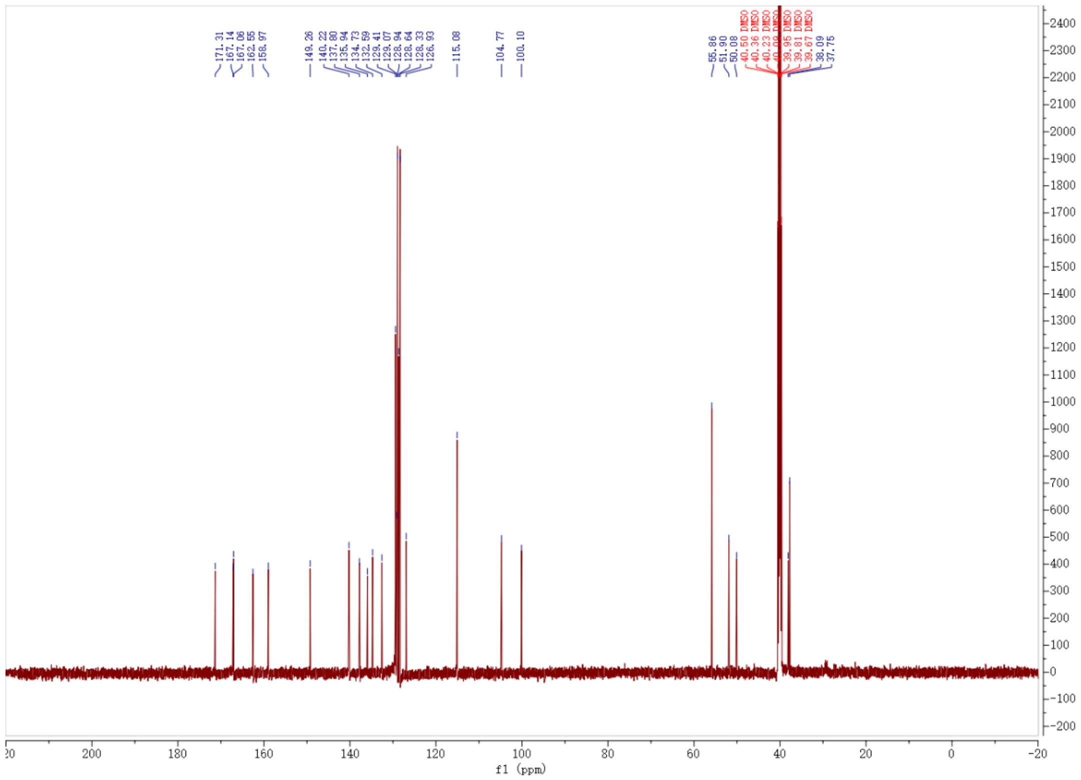

171.31
167.14
167.06
162.55
158.97
149.26
140.22
137.80
135.94
134.73
132.59
129.41
129.07
128.94
128.64
128.33
126.93
115.08
104.77
100.10
55.86
51.90
50.08
40.50 DMSO
40.36 DMSO
40.23 DMSO
39.95 DMSO
39.81 DMSO
39.67 DMSO
38.09
37.75
f1 (ppm)

1.4 MS, ^{1}H-NMR and ^{13}C-NMR Spectra for TD-1b

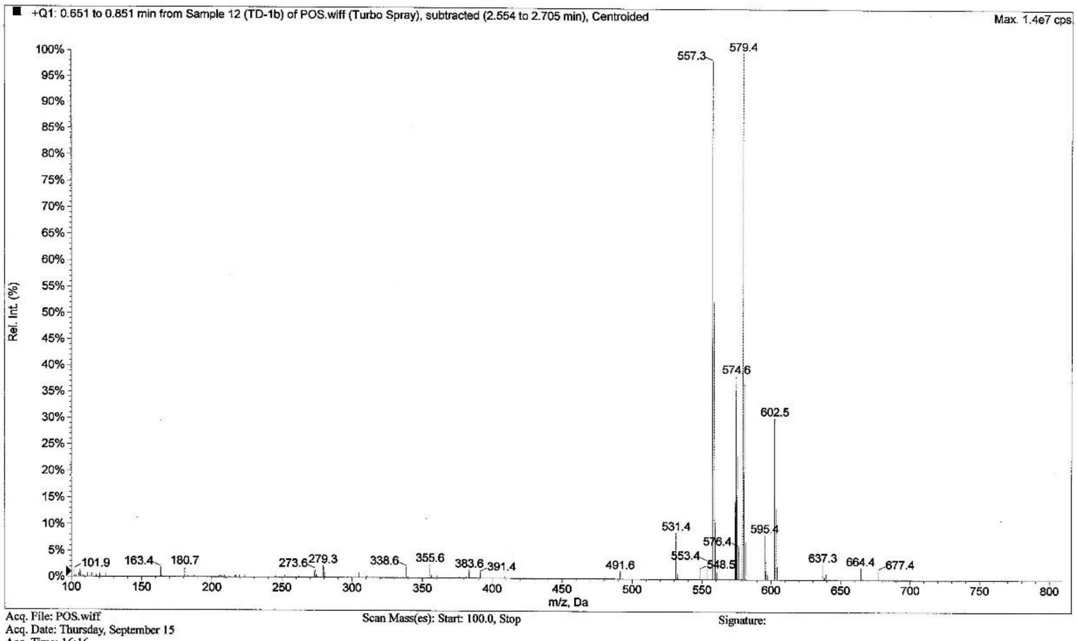

Acq. File: POS.wiff
Acq. Date: Thursday, September 15
Acq. Time: 16:16
Batch Name: 20220915.dab
Sample Number: N/A
Sample Name: TD-1b

Scan Mass(es): Start: 100.0, Stop

Collision Energy: N/A
Ion Energy: N/A

Signature:

Drug Analysis Center
School of Pharmaceutical Science
Shandong University

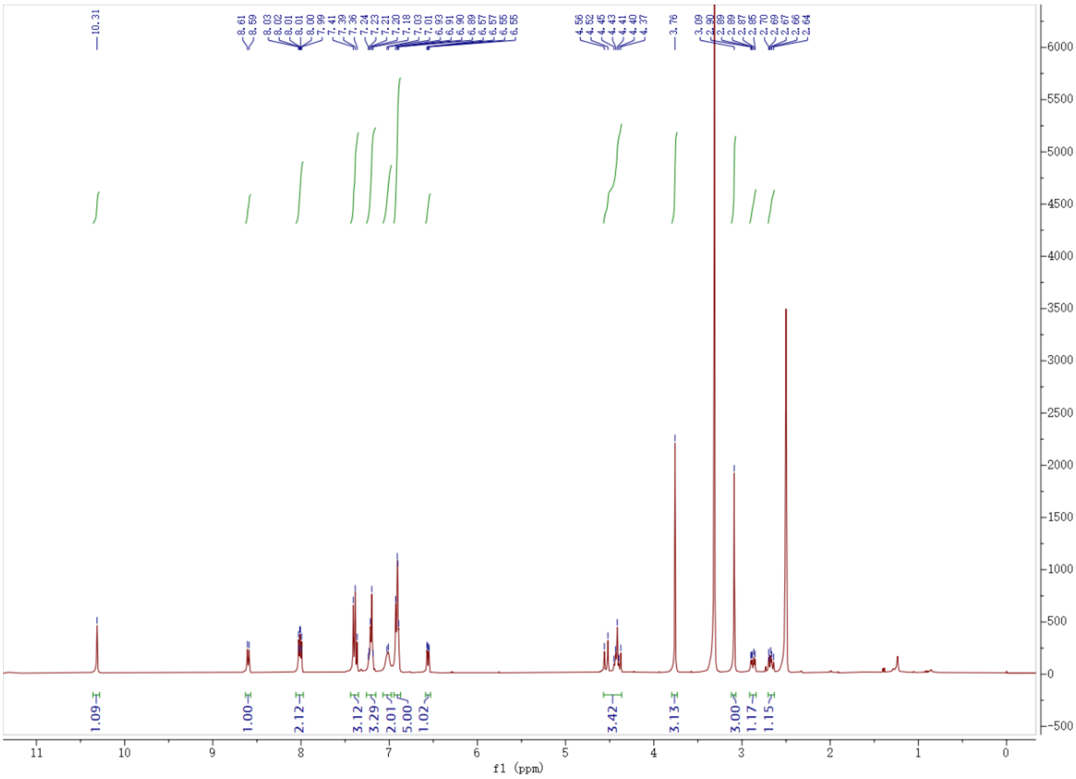

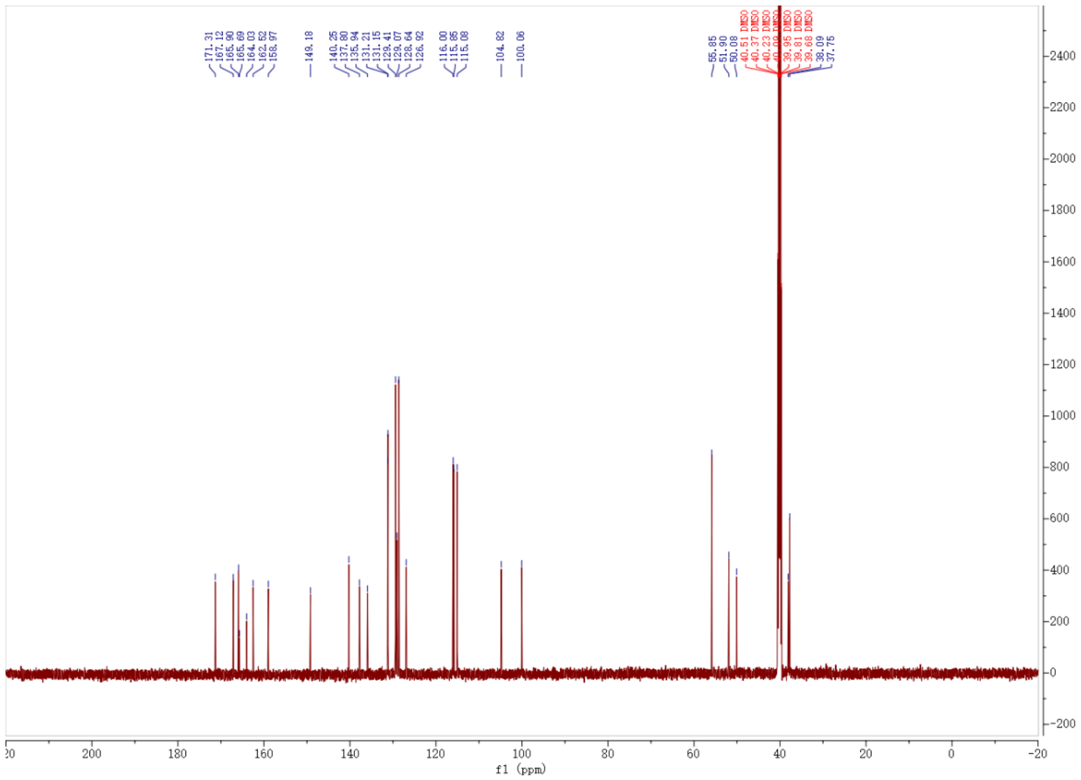
171.31
167.12
165.90
165.69
164.03
162.52
158.97
149.18
140.25
137.80
135.94
131.21
131.15
129.41
129.07
128.64
126.92
116.00
115.85
115.08
104.82
100.06
55.85
51.90
50.08
40.51 DMSO
40.37 DMSO
40.23 DMSO
40.09 DMSO
39.95 DMSO
39.81 DMSO
39.68 DMSO
38.09
37.75
2400
2200
2000
1800
1600
1400
1200
1000
800
600
400
200
0
-200
200
180
160
140
120
100
80
60
40
20
0
-20
f1 (ppm)

1.5 MS, ^{1}H-NMR and ^{13}C-NMR Spectra for TD-1c

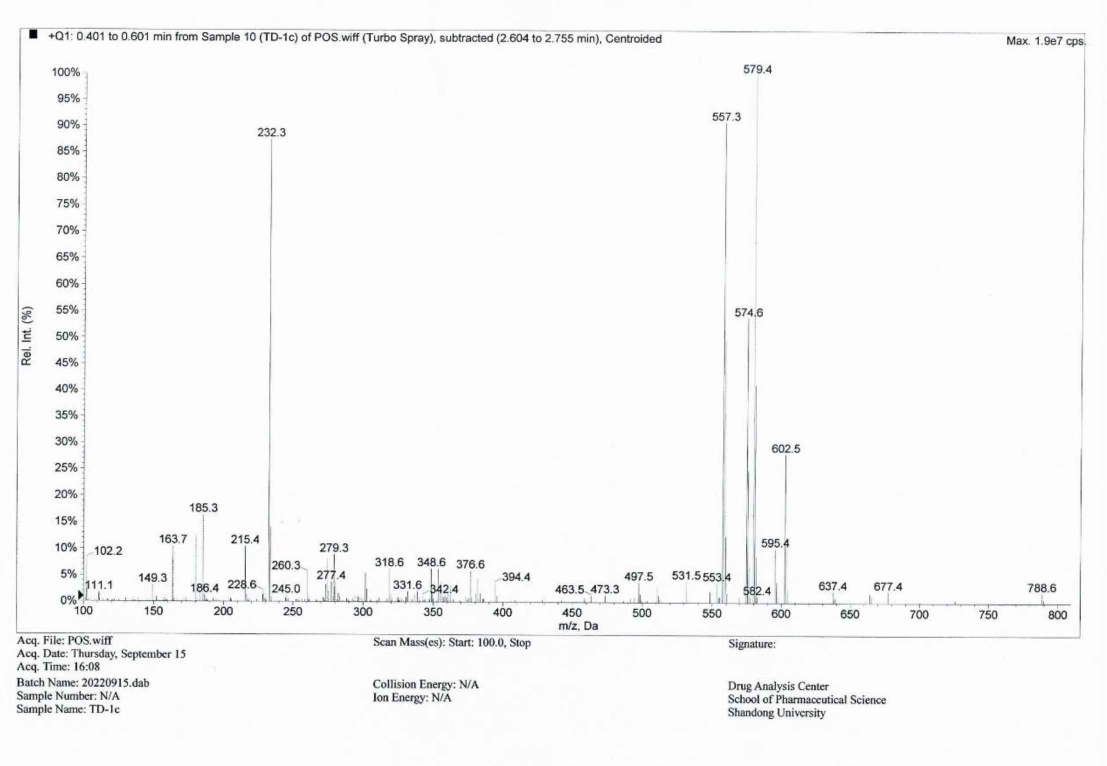

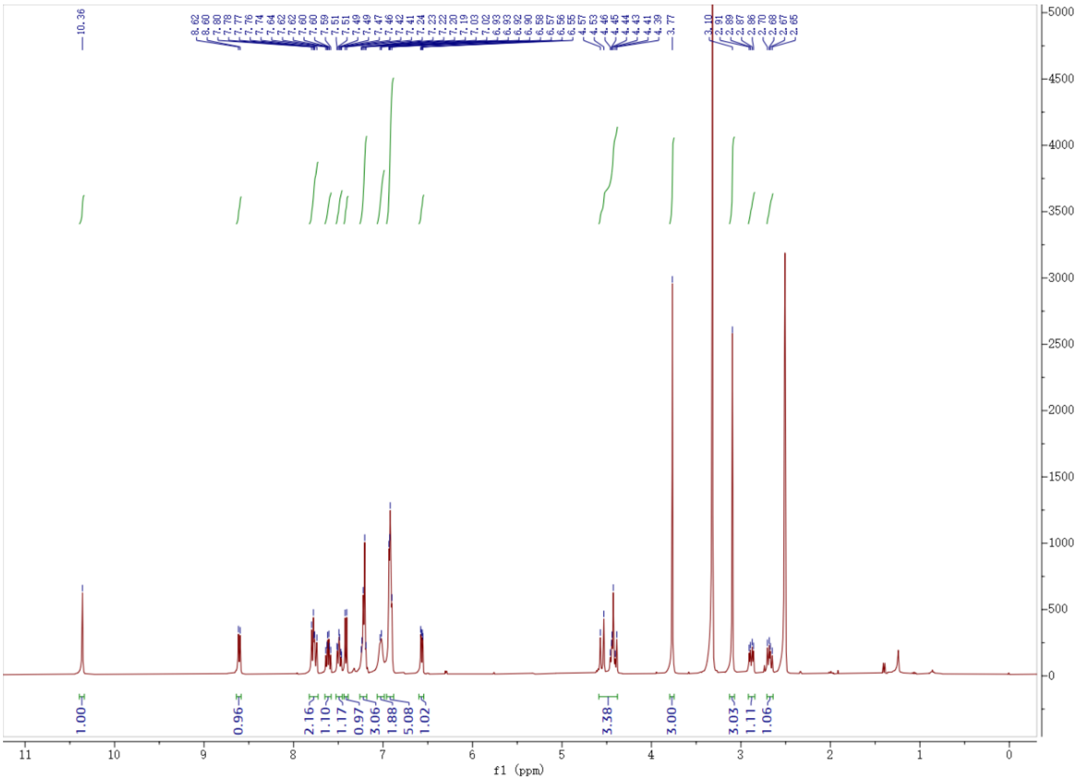

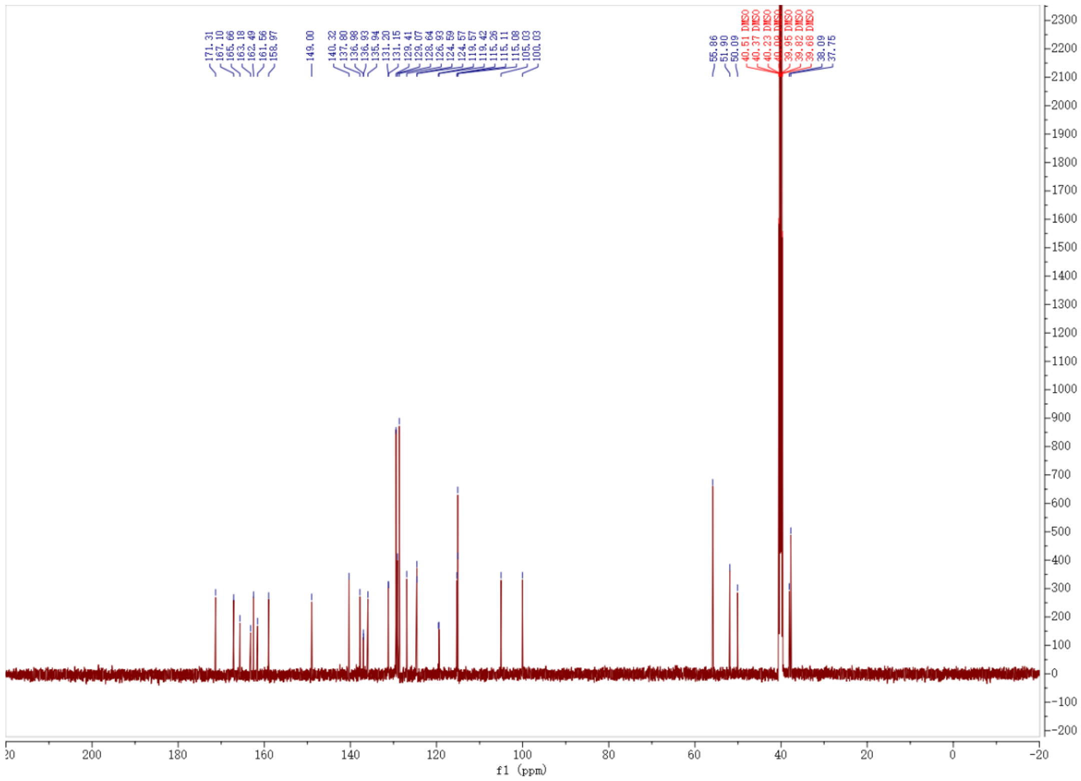
171.31
167.10
165.66
163.18
162.49
161.56
158.97
149.00
140.32
137.80
136.98
136.93
135.94
131.20
131.15
129.41
129.07
128.64
126.93
124.59
124.57
119.57
119.42
115.26
115.11
115.08
105.03
100.03
55.86
51.90
50.09
40.51 DMSO
40.37 DMSO
40.23 DMSO
40.09 DMSO
39.95 DMSO
39.82 DMSO
39.68 DMSO
38.09
37.75
f1 (ppm)

1.6 MS, ^{1}H-NMR and ^{13}C-NMR Spectra for TD-1d

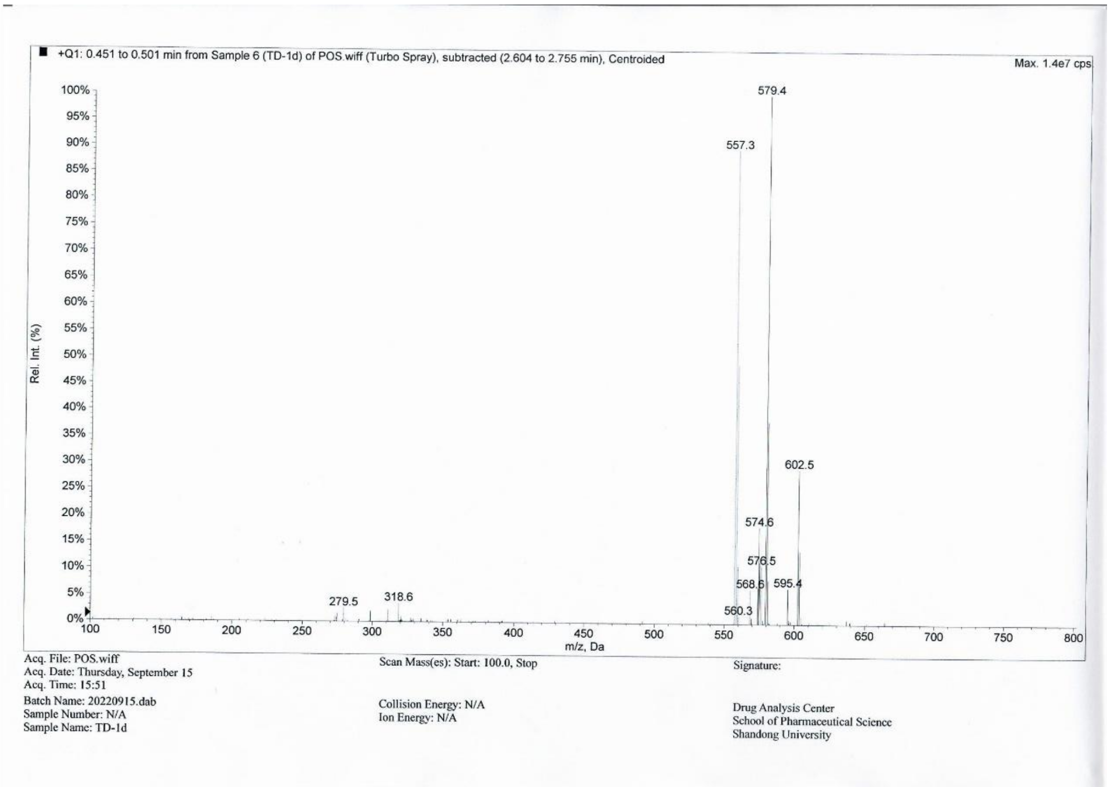

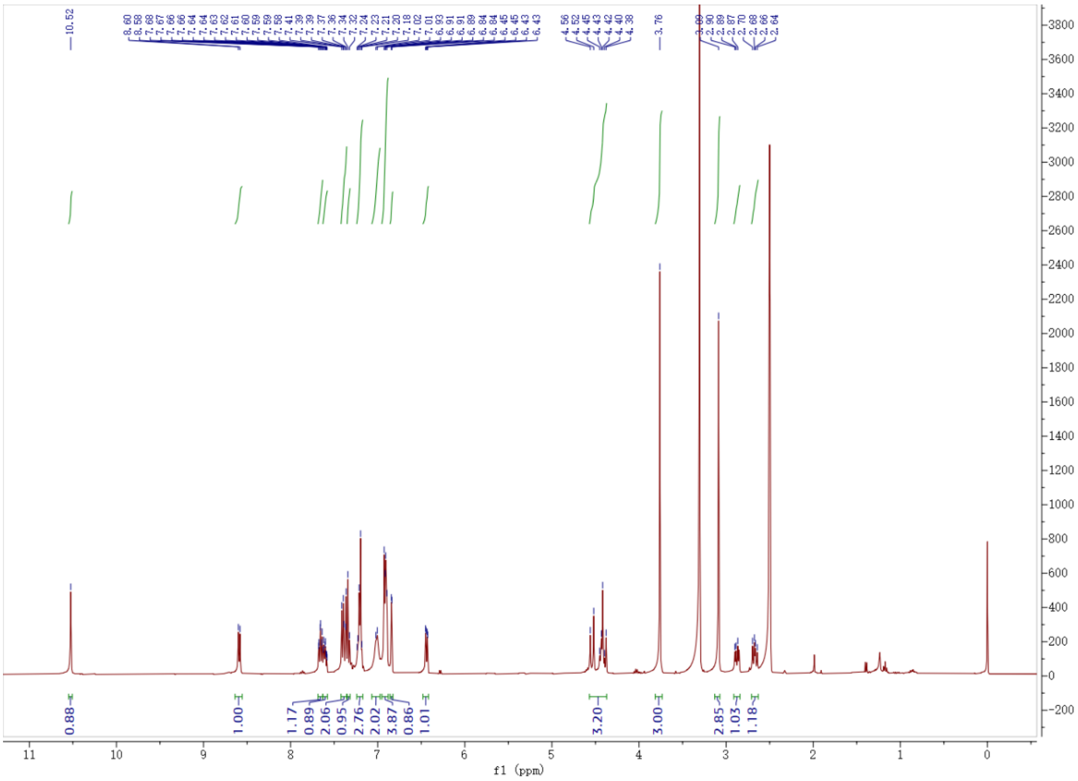

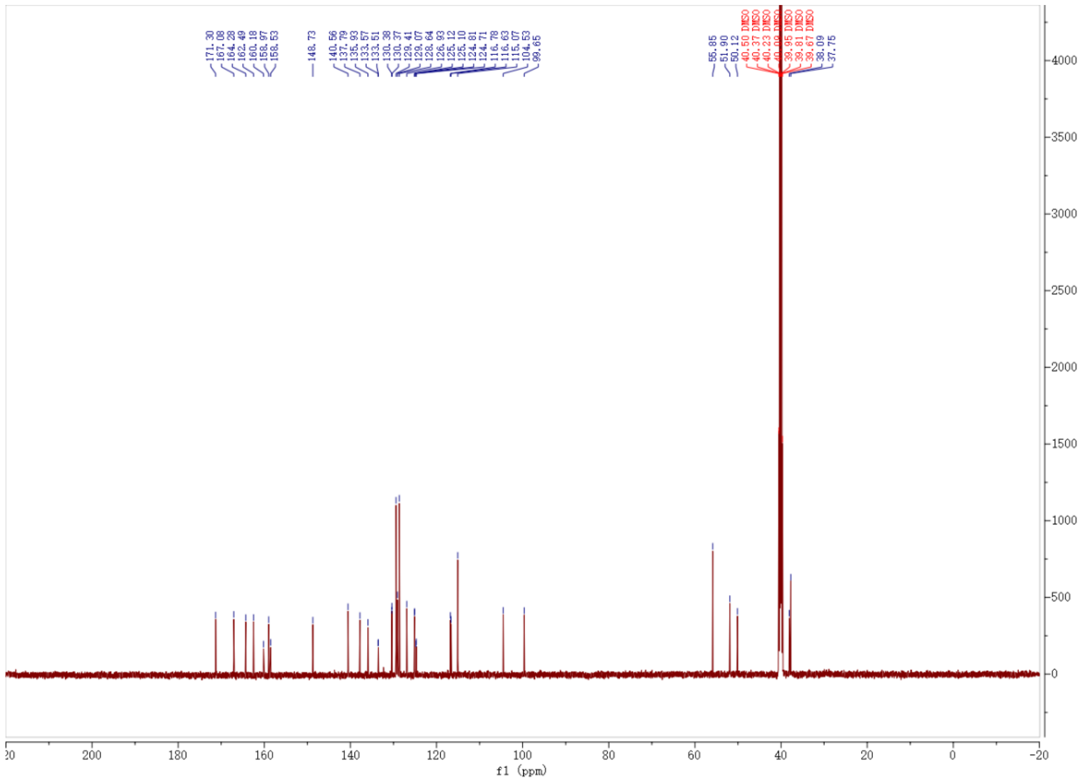
171.30
167.08
164.28
162.49
160.18
158.97
158.53
148.73
140.56
137.79
135.93
133.57
133.51
130.38
130.37
129.41
129.07
128.64
126.93
125.12
125.10
124.81
124.71
116.78
116.63
115.07
104.53
99.65
55.85
51.90
50.12
40.50 DMSO
40.37 DMSO
40.23 DMSO
39.95 DMSO
39.81 DMSO
39.67 DMSO
38.09
37.75
f1 (ppm)

1.7 MS, ^{1}H-NMR and ^{13}C-NMR Spectra for TD-1e

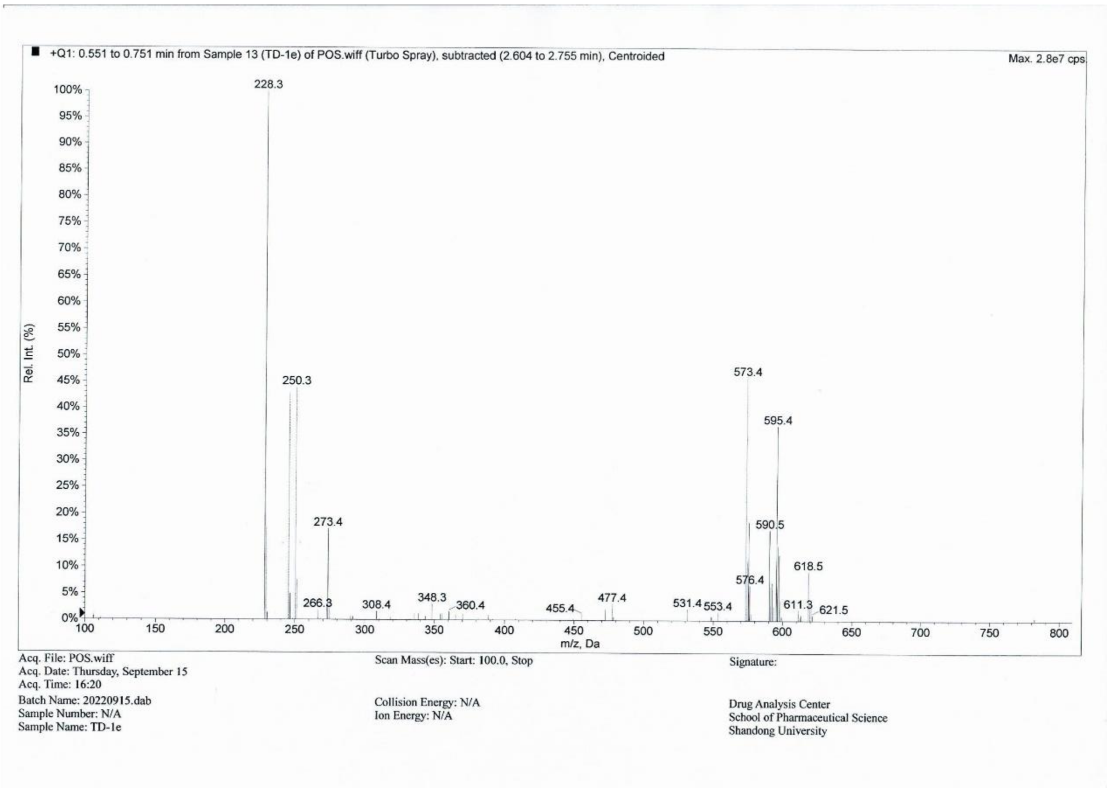

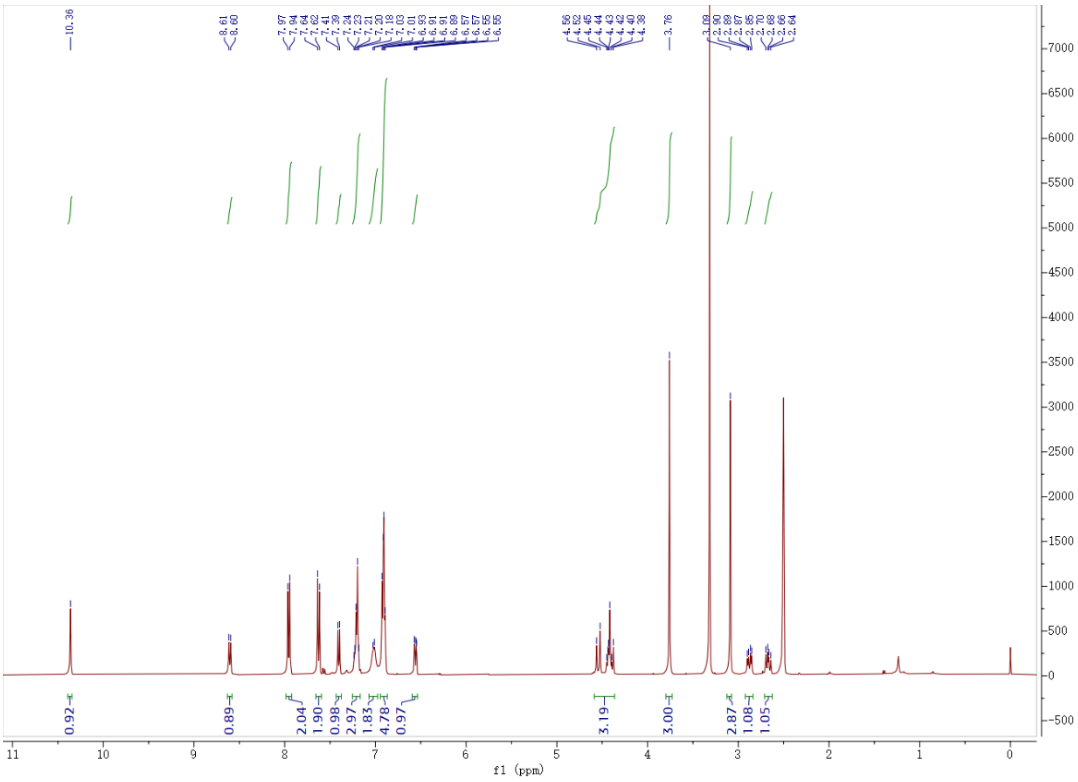

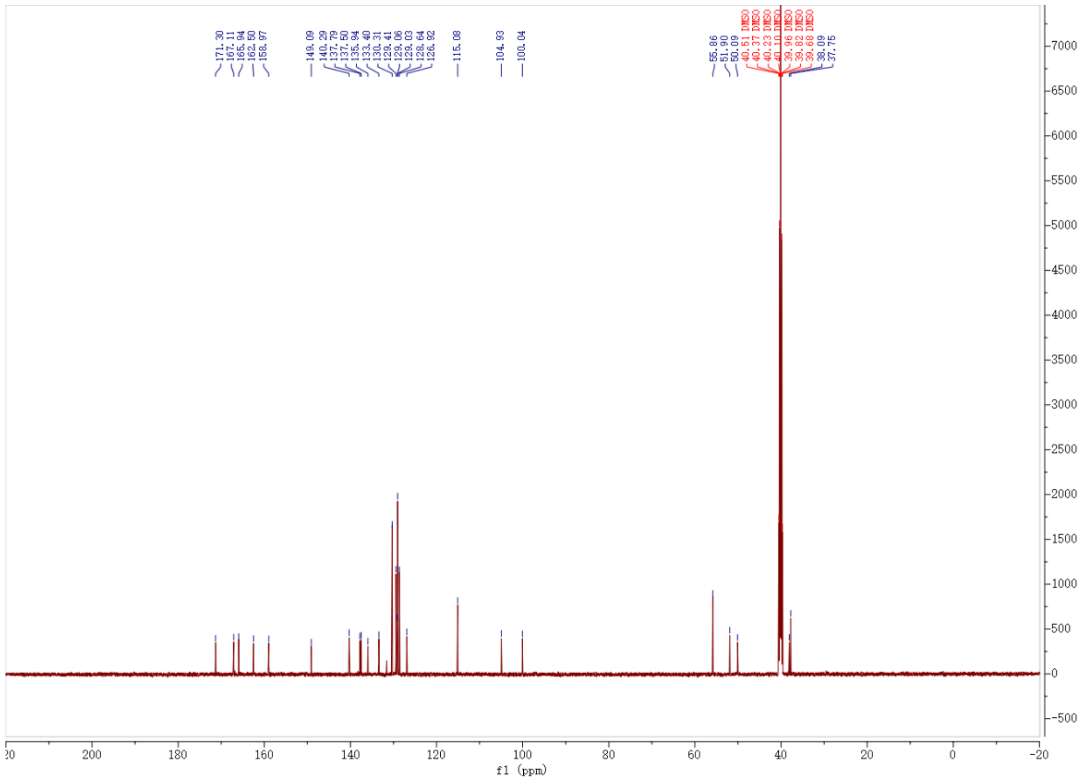

171.30
167.11
165.94
162.50
158.97
149.09
140.29
137.79
137.50
135.94
133.40
130.31
129.41
129.06
129.03
128.64
126.92
115.08
104.93
100.04
55.86
51.90
50.09
40.51 DMSO
40.37 DMSO
40.23 DMSO
40.10 DMSO
39.96 DMSO
39.82 DMSO
39.68 DMSO
38.09
37.75
f1 (ppm)

1.8 MS, ^{1}H-NMR and ^{13}C-NMR Spectra for TD-1f

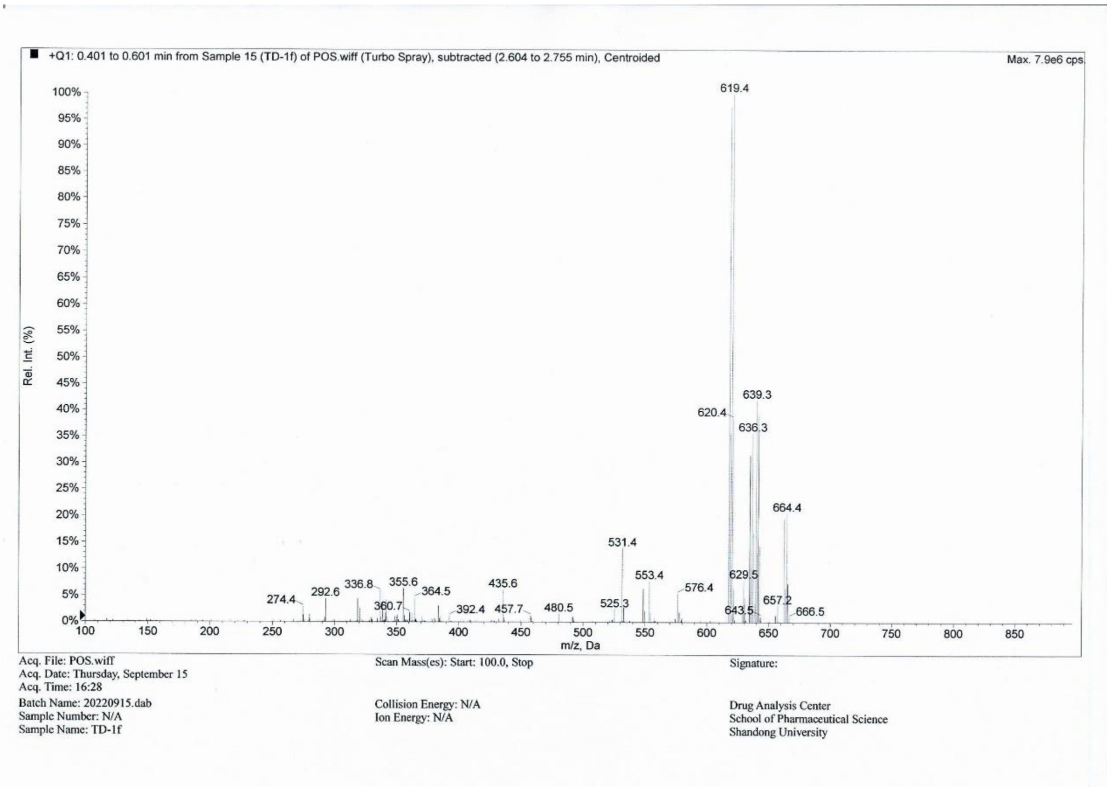

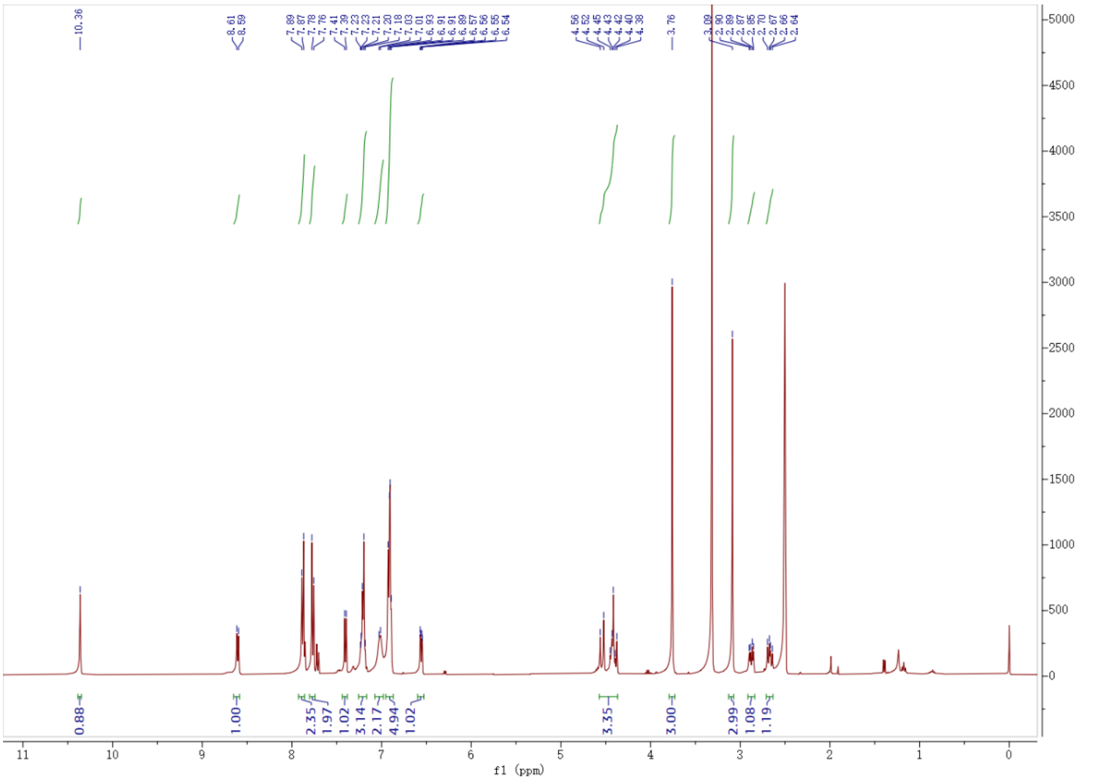

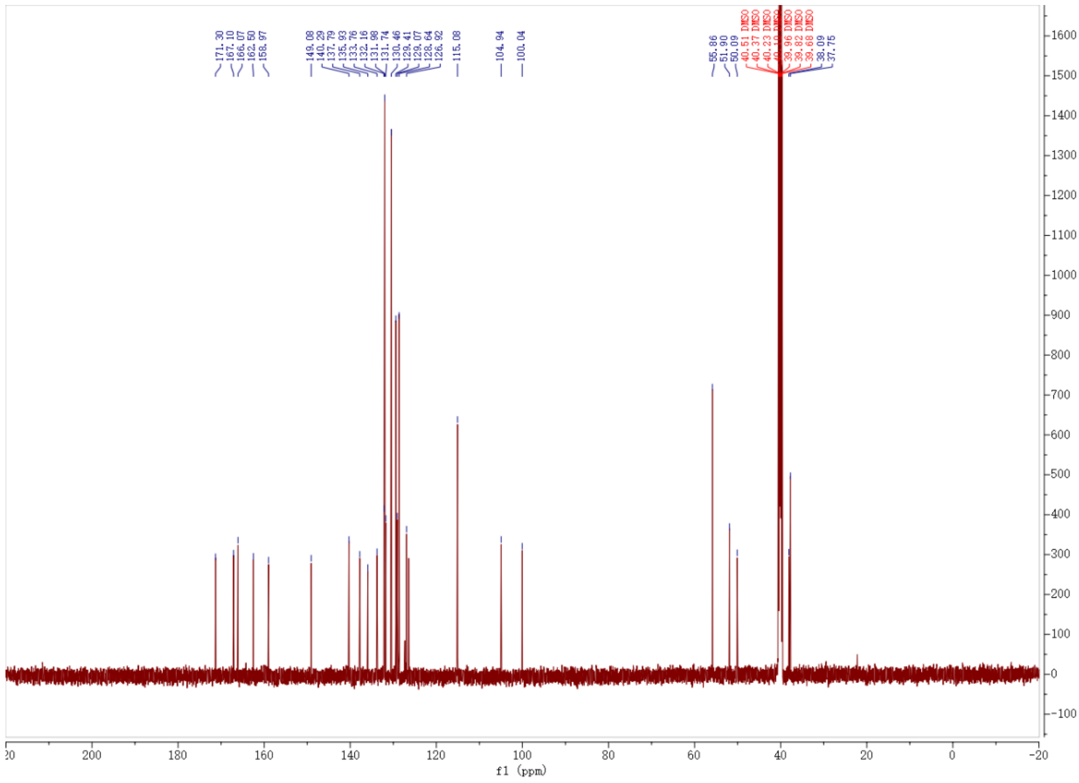
171.30
167.10
166.07
162.50
158.97
149.08
140.29
137.79
135.93
133.76
132.16
131.98
131.74
130.46
129.41
129.07
128.64
126.92
115.08
104.94
100.04
55.86
51.90
50.09
40.51 DMSO
40.37 DMSO
40.23 DMSO
40.10 DMSO
39.96 DMSO
39.82 DMSO
39.68 DMSO
38.09
37.75
f1 (ppm)

1.9 MS, ^{1}H-NMR and ^{13}C-NMR Spectra for TD-1g

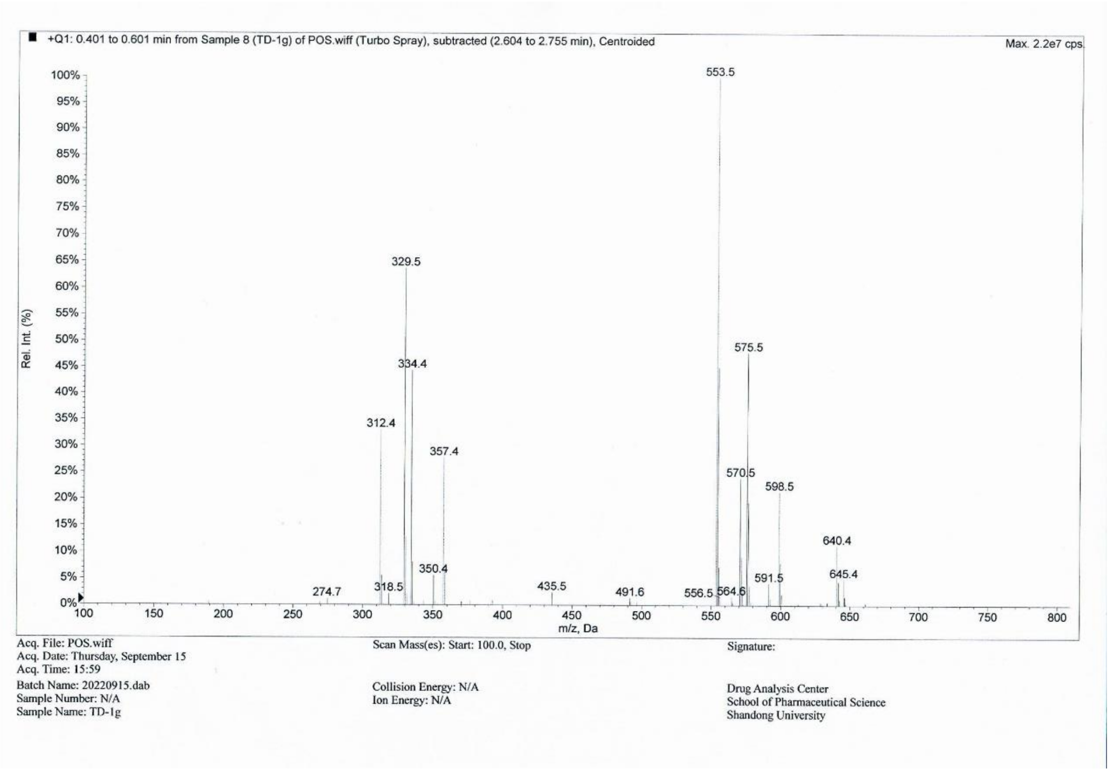

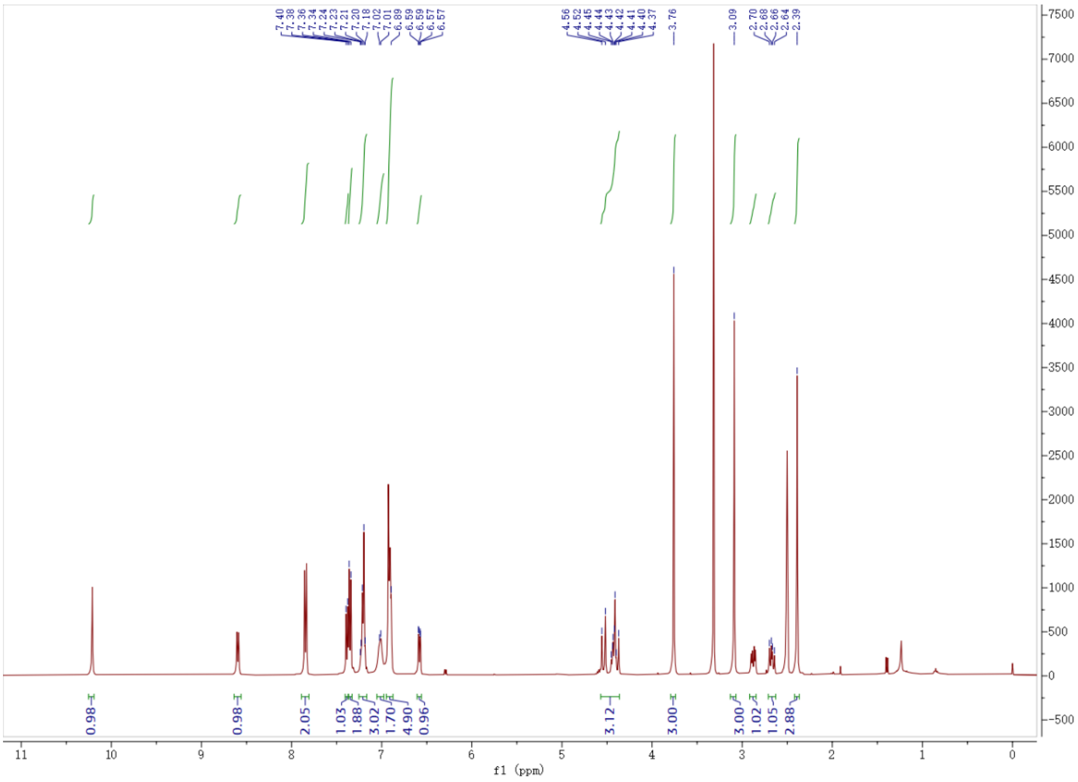

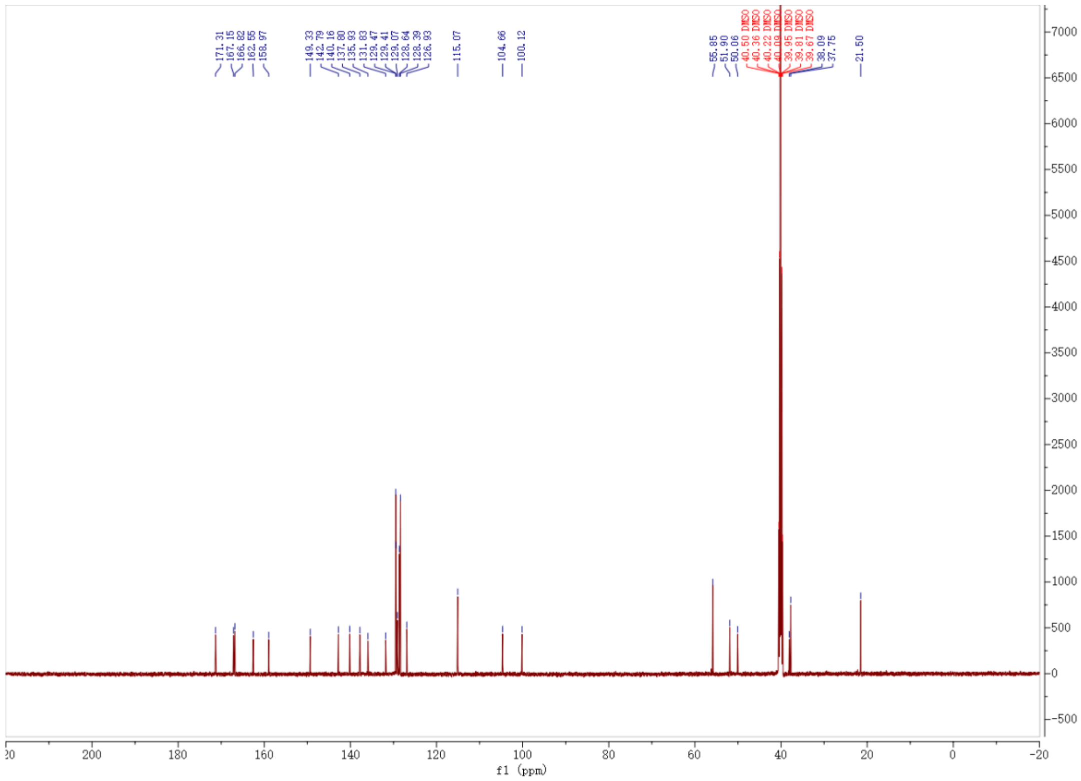

171.31
167.15
166.82
162.55
158.97
149.33
142.79
140.16
137.80
135.93
131.83
129.47
129.41
129.07
128.64
128.39
126.93
115.07
104.66
100.12
55.85
51.90
50.06
40.50 DMSO
40.36 DMSO
40.22 DMSO
39.95 DMSO
39.81 DMSO
39.67 DMSO
38.09
37.75
21.50
f1 (ppm)

1.10 MS, ^{1}H-NMR and ^{13}C-NMR Spectra for TD-1h

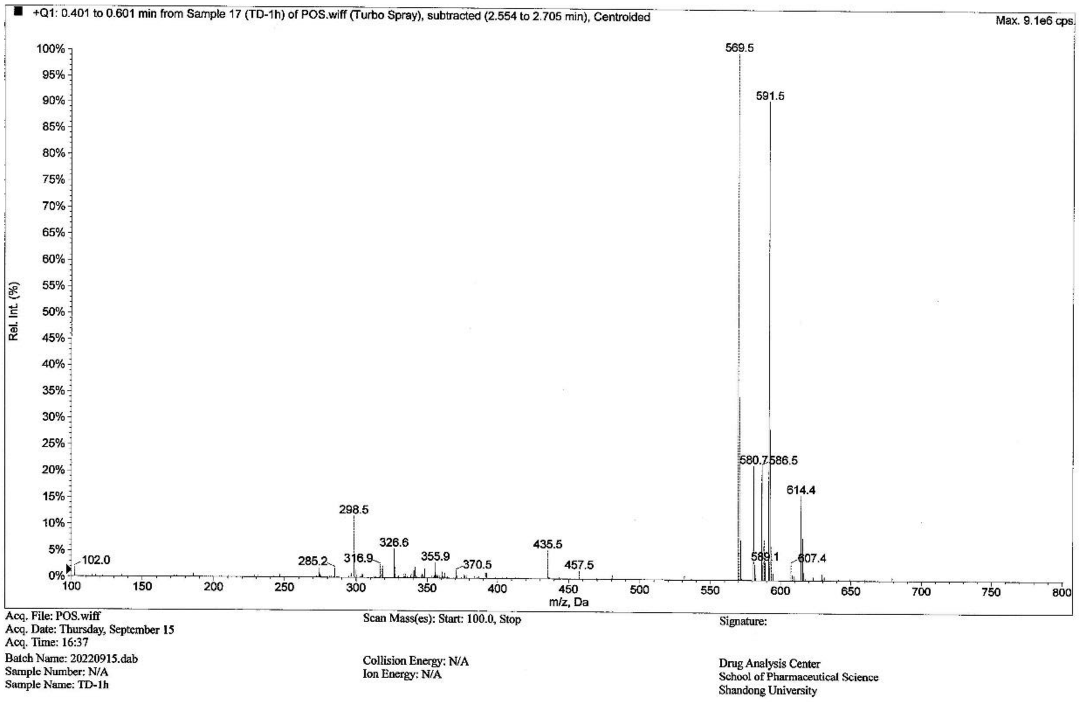

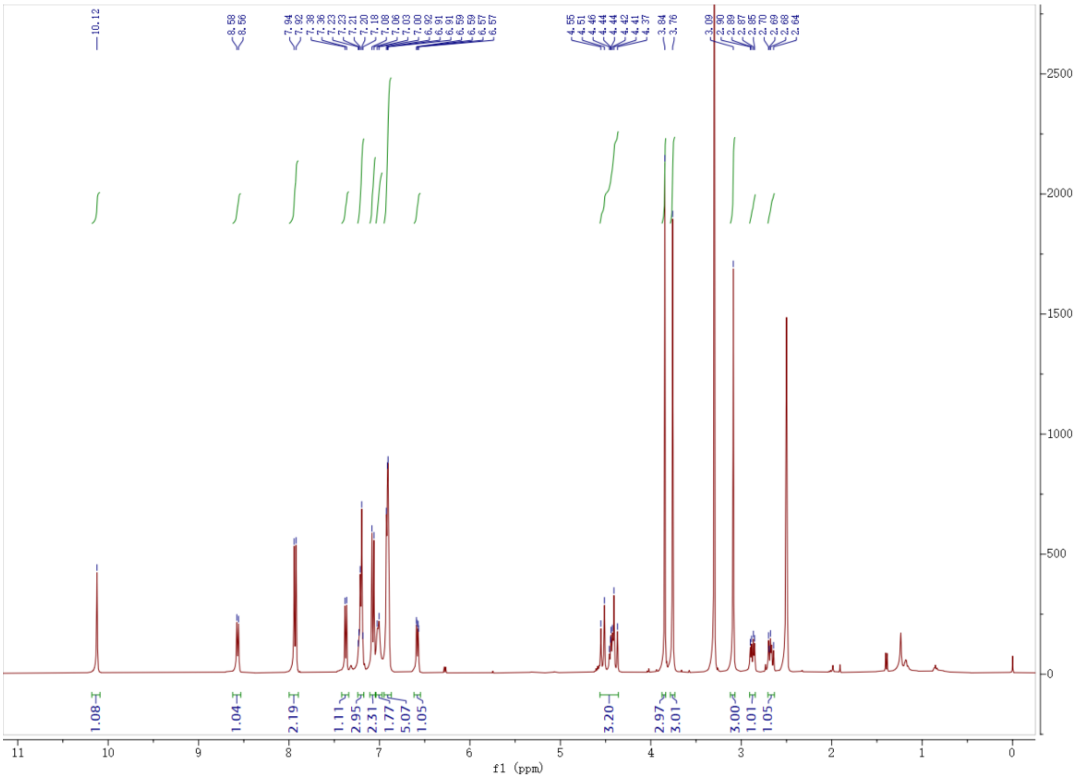

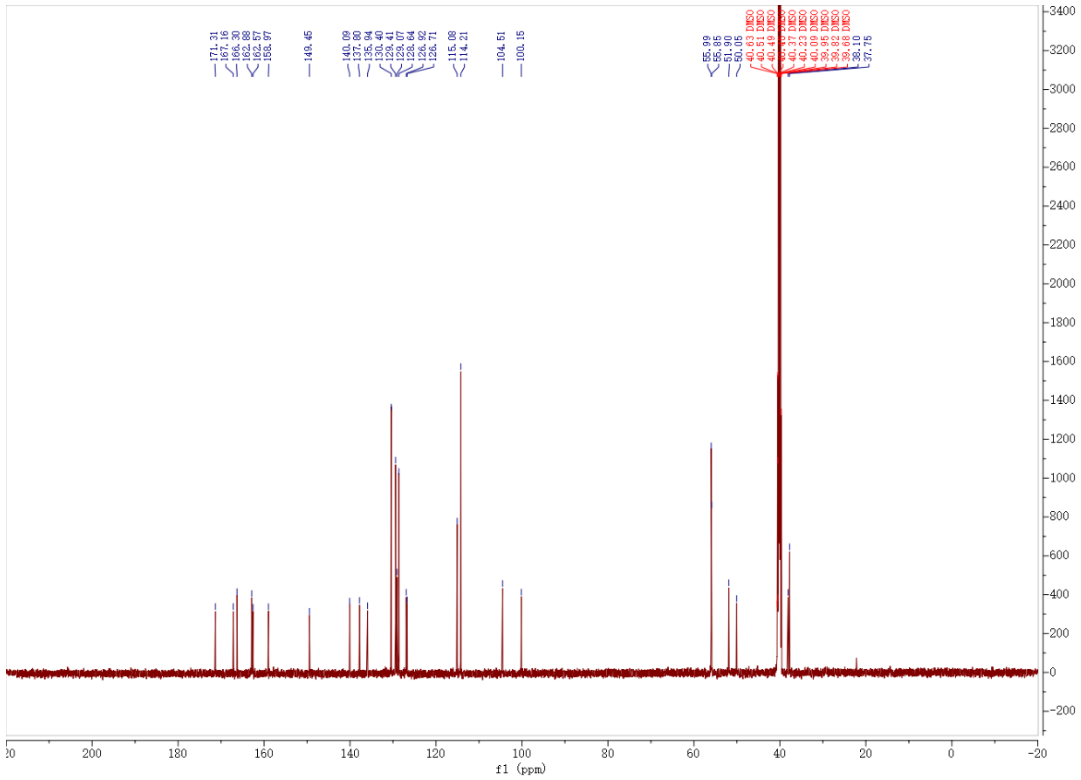
171.31
167.16
166.30
162.88
162.57
158.97
149.45
140.09
137.80
135.94
130.40
129.41
129.07
128.64
126.92
126.71
115.08
114.21
104.51
100.15
55.99
55.85
51.90
50.05
40.63 DMSO
40.51 DMSO
40.49 DMSO
40.37 DMSO
40.23 DMSO
40.09 DMSO
39.95 DMSO
39.82 DMSO
39.68 DMSO
38.10
37.75
f1 (ppm)

1.11 MS, ^{1}H-NMR and ^{13}C-NMR Spectra for TD-1i

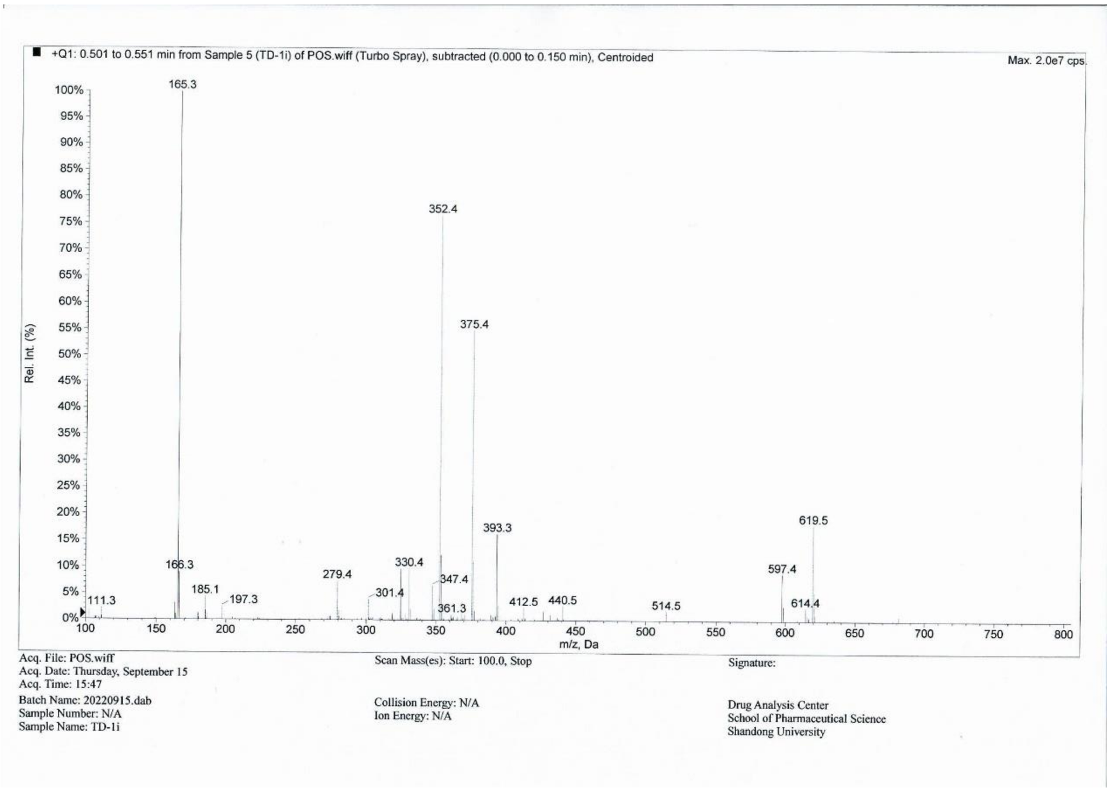

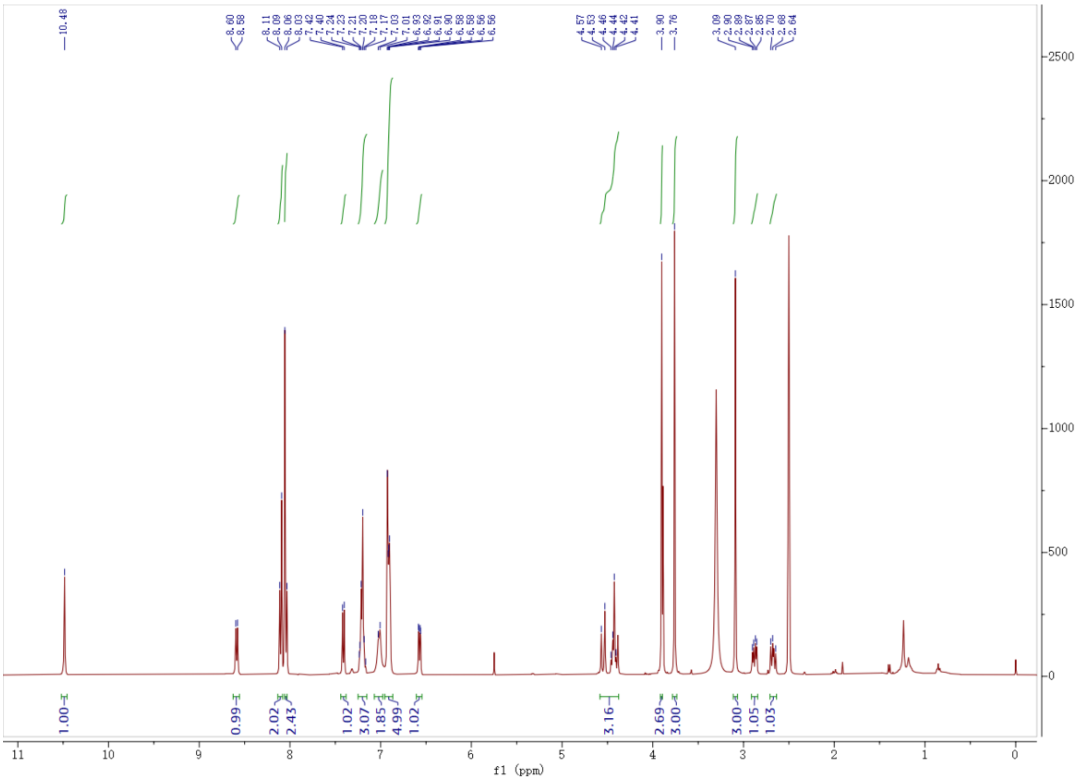

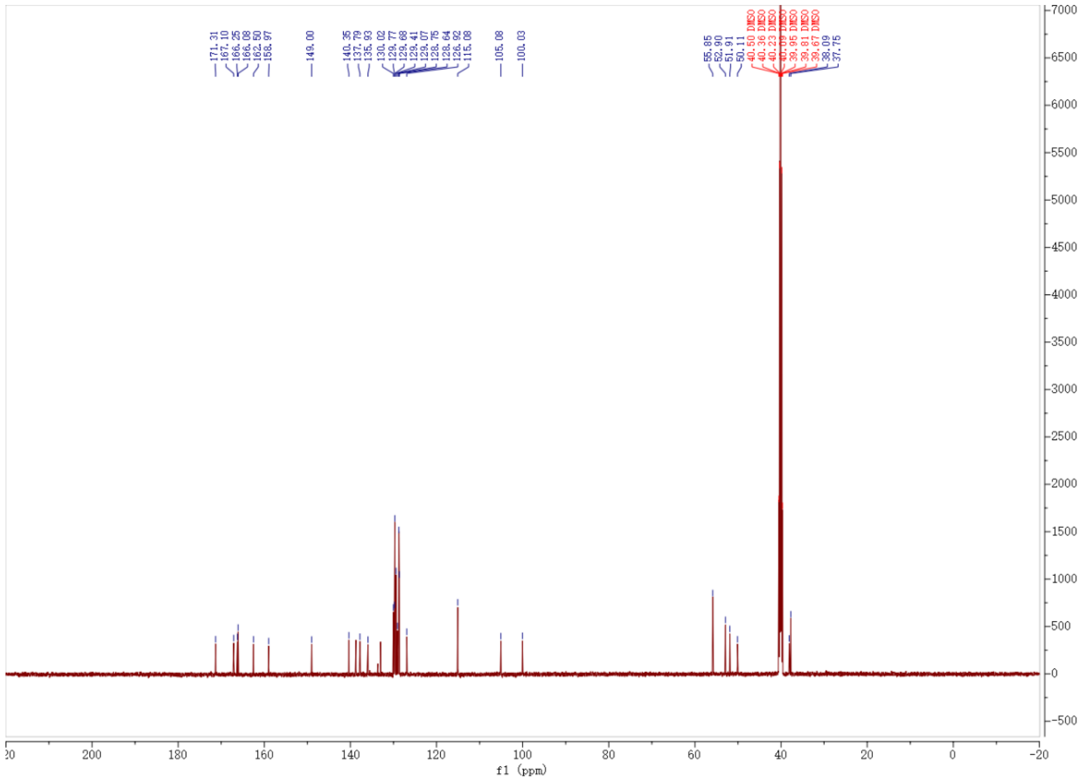
171.31
167.10
166.25
166.08
162.50
158.97
149.00
140.35
137.79
135.93
130.02
129.77
129.68
129.41
129.07
128.75
128.64
126.92
115.08
105.08
100.03
55.85
52.90
51.91
50.11
40.50 DMSO
40.36 DMSO
40.23 DMSO
40.09 DMSO
39.95 DMSO
39.81 DMSO
39.67 DMSO
38.09
37.75
7000
6500
6000
5500
5000
4500
4000
3500
3000
2500
2000
1500
1000
500
0
-500
200
180
160
140
120
100
80
60
40
20
0
-20
f1 (ppm)

1.12 MS, ^{1}H-NMR and ^{13}C-NMR Spectra for TD-1j

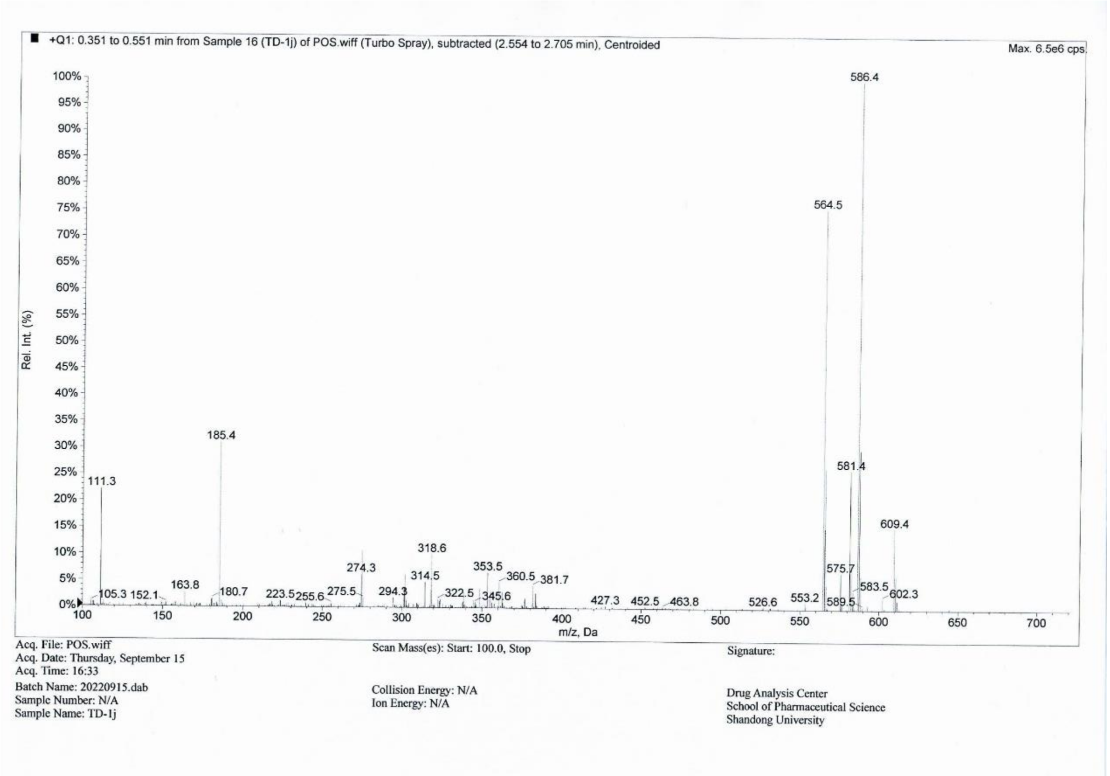

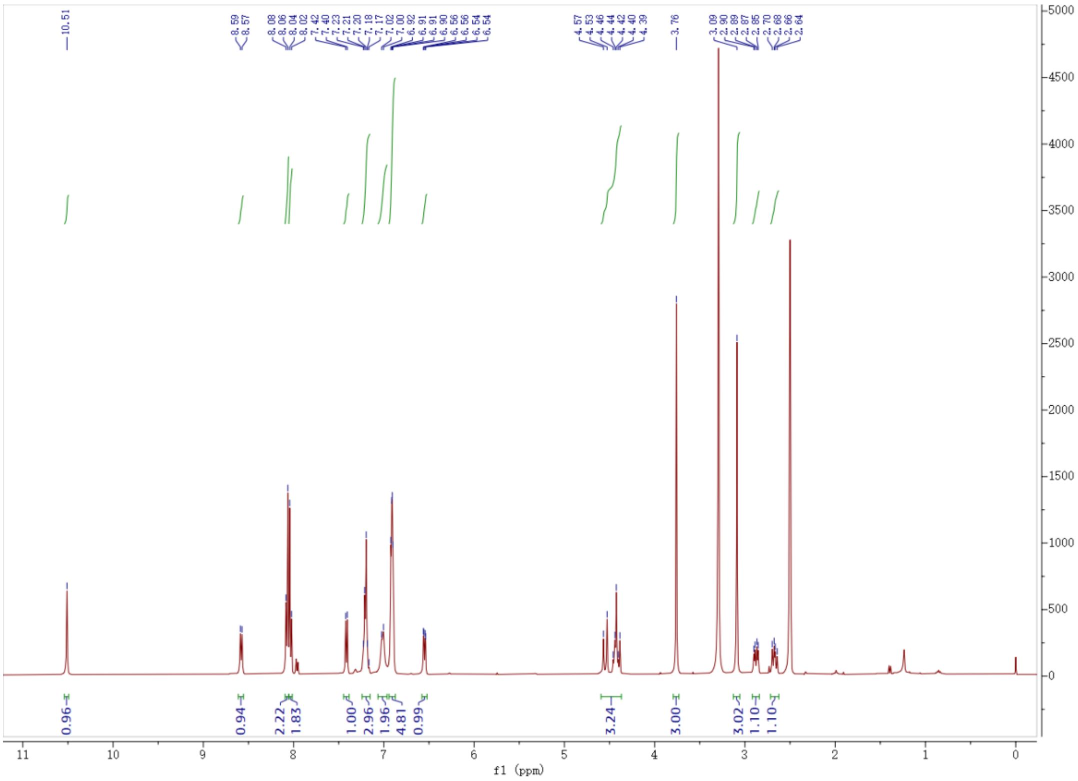

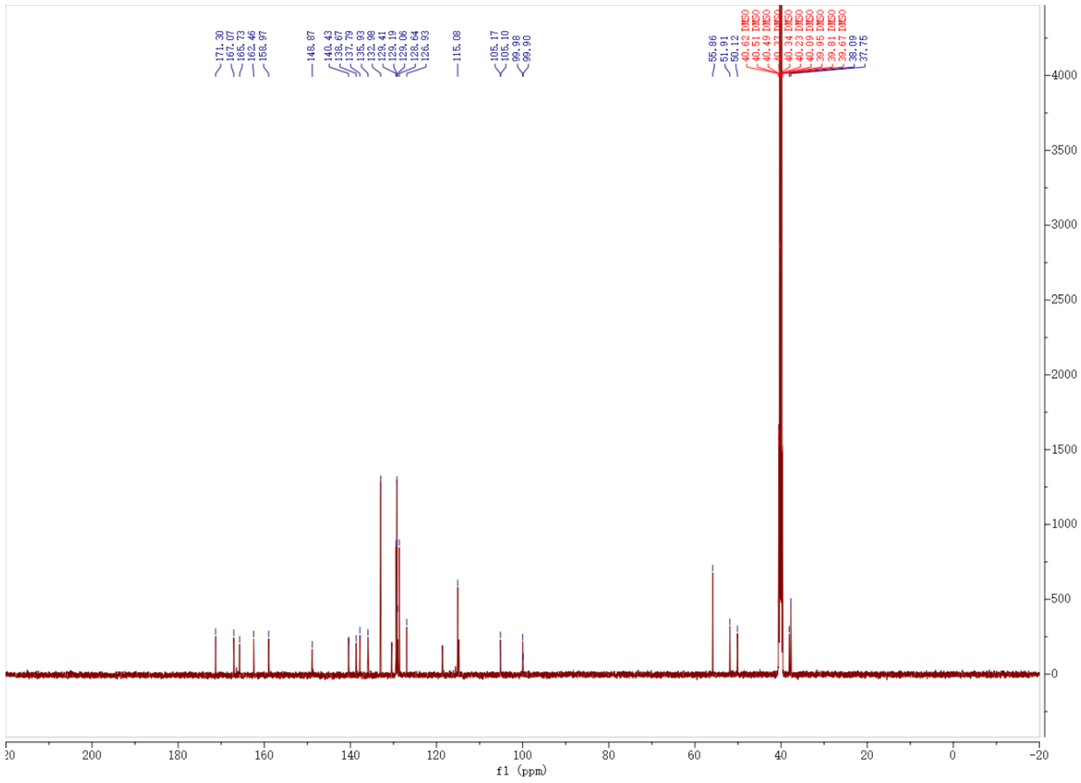

171.30
167.07
165.73
162.46
158.97
148.87
140.43
138.67
137.79
135.93
132.98
129.41
129.19
129.06
128.64
126.93
115.08
105.17
105.10
99.98
99.90
55.86
51.91
50.12
40.62 DMSO
40.51 DMSO
40.49 DMSO
40.37 DMSO
40.34 DMSO
40.23 DMSO
40.09 DMSO
39.95 DMSO
39.81 DMSO
39.67 DMSO
38.09
37.75
f1 (ppm)

1.13 MS, ^{1}H-NMR and ^{13}C-NMR Spectra for TD-1k

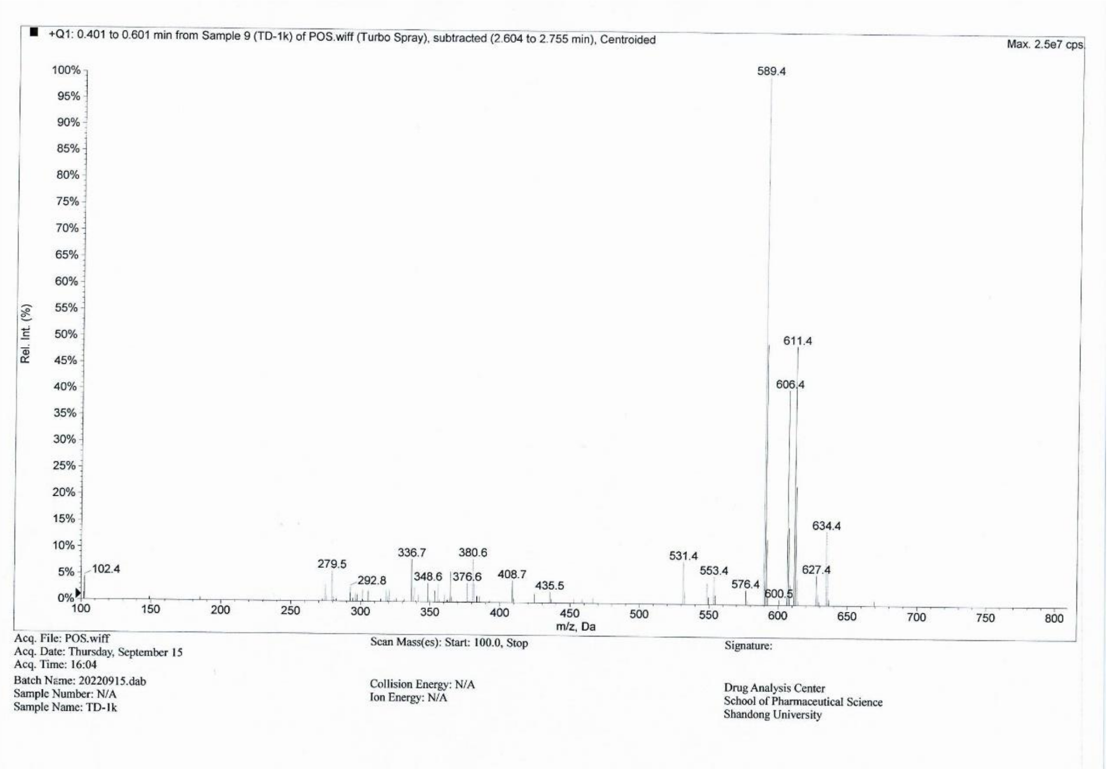

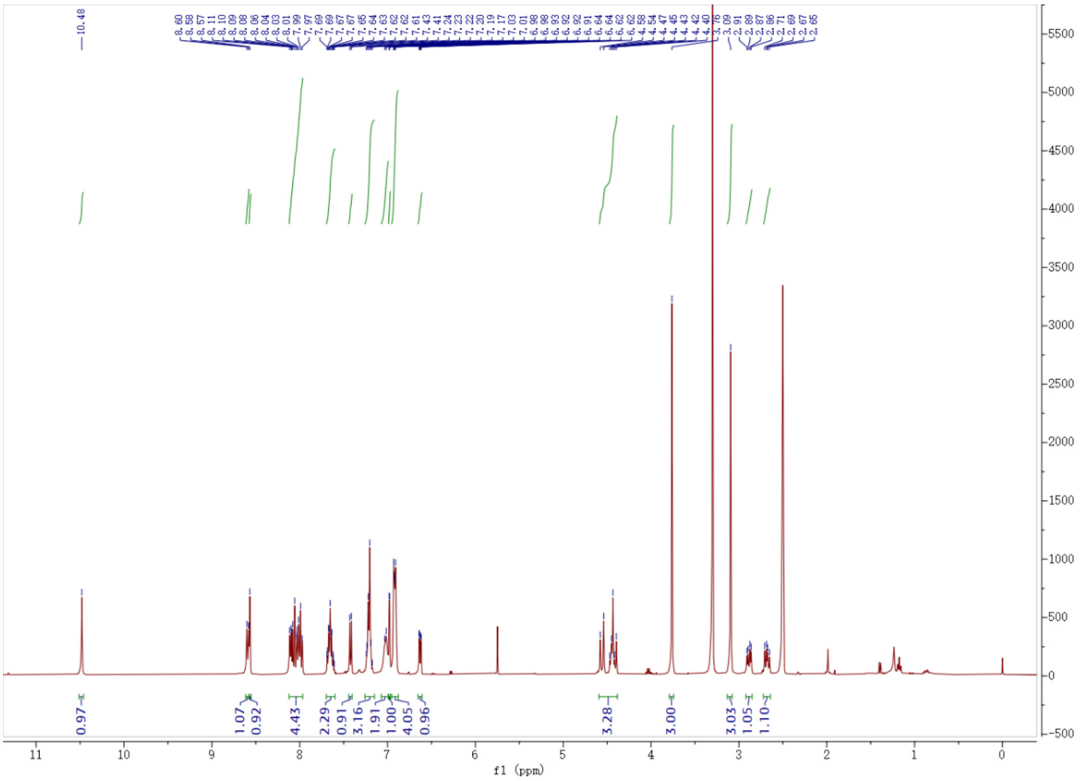

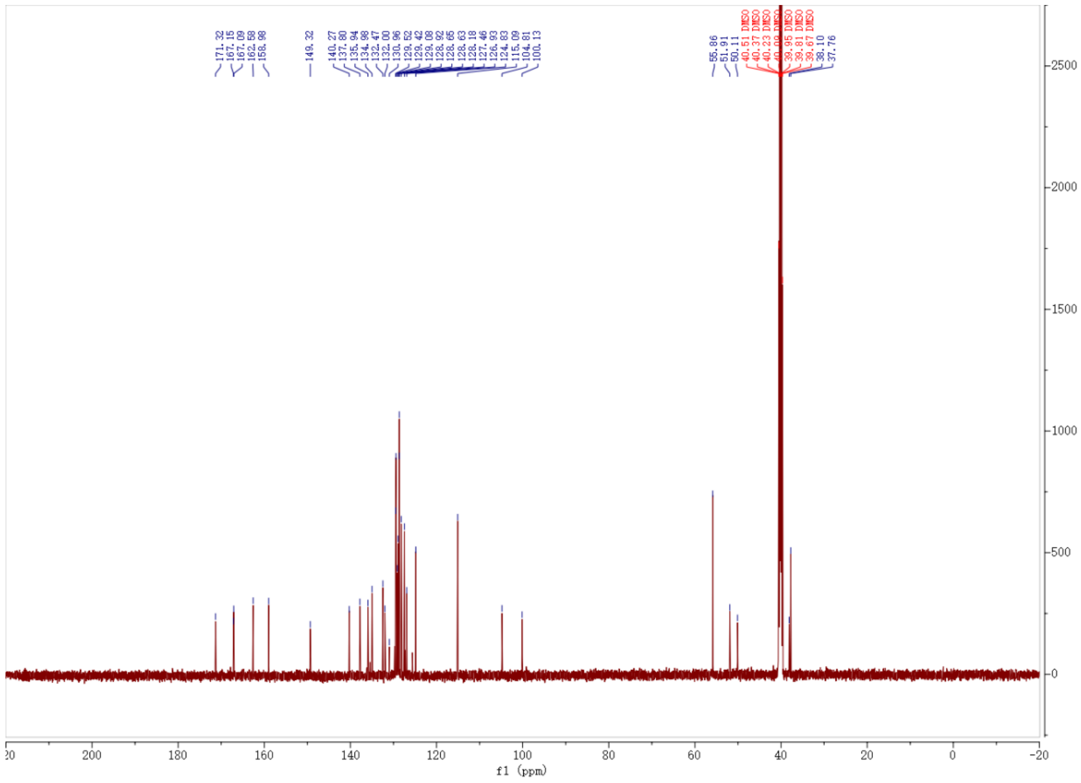
171.32
167.15
167.09
162.58
158.98
149.32
140.27
137.80
135.94
134.98
132.47
132.00
130.96
129.52
129.42
129.08
128.92
128.65
128.63
128.18
127.46
126.93
124.83
115.09
104.81
100.13
55.86
51.91
50.11
40.51 DMSO
40.37 DMSO
40.23 DMSO
40.09 DMSO
39.95 DMSO
39.81 DMSO
39.67 DMSO
38.10
37.76
2500
2000
1500
1000
500
0
200
180
160
140
120
100
80
60
40
20
0
-20
f1 (ppm)

1.14 MS, ^{1}H-NMR and ^{13}C-NMR Spectra for TD-1l

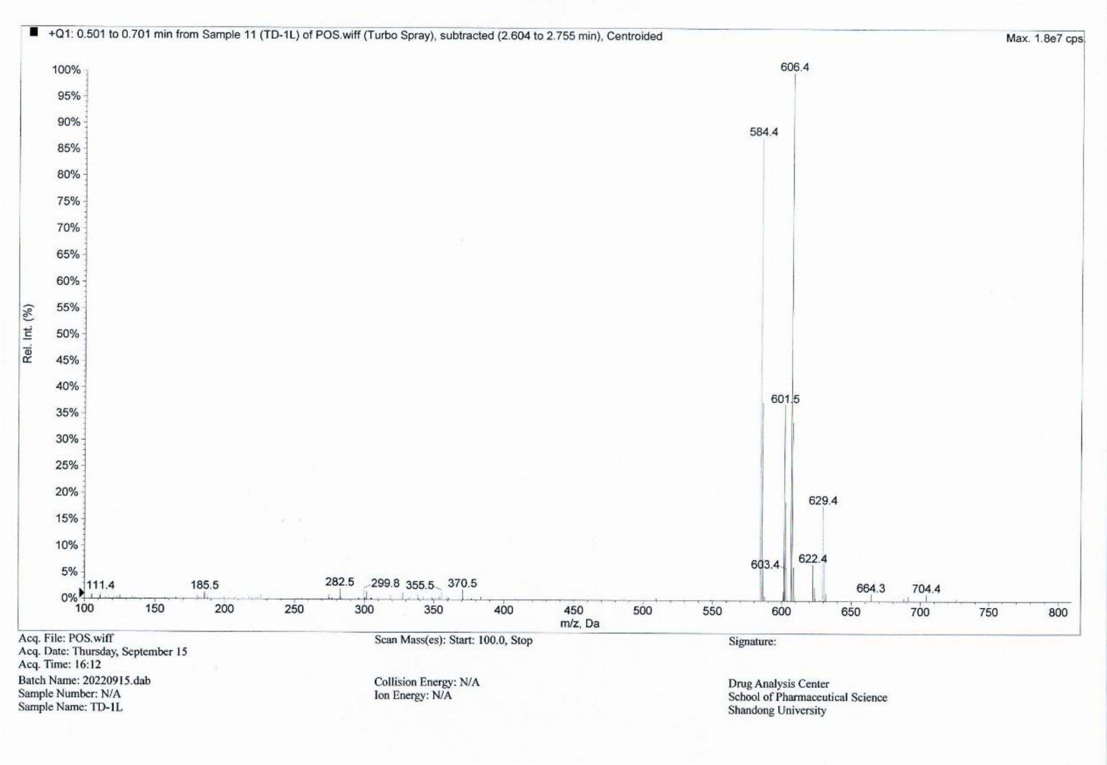

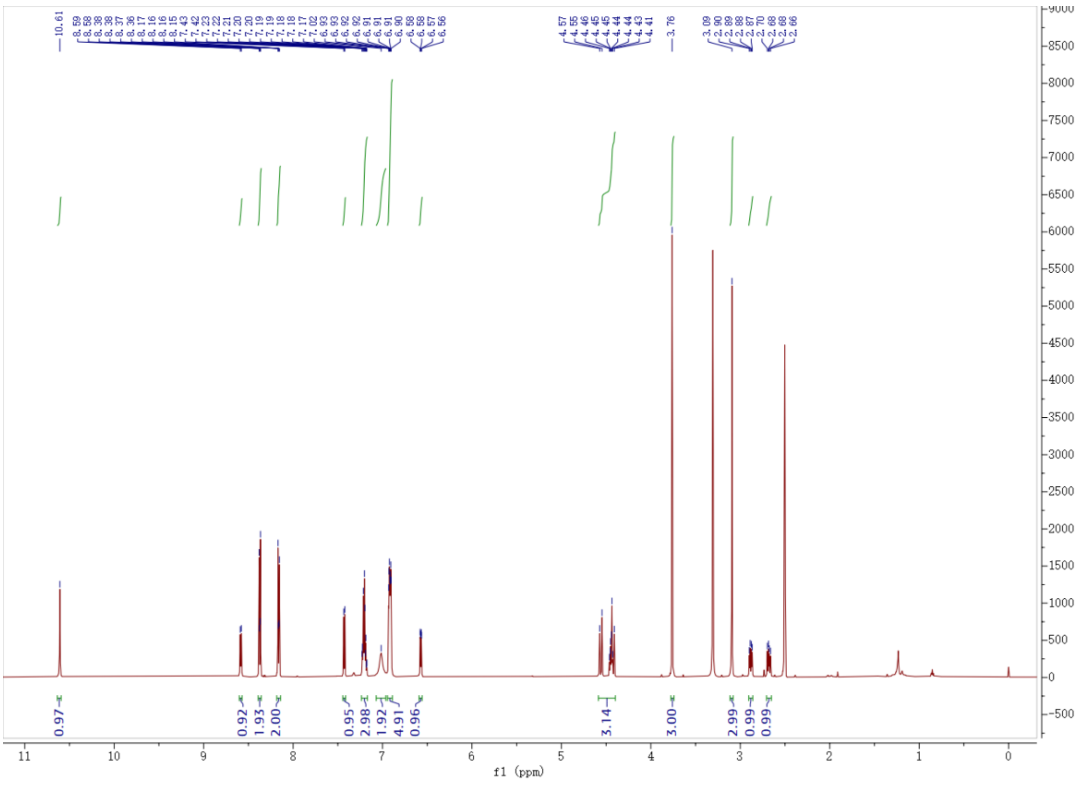

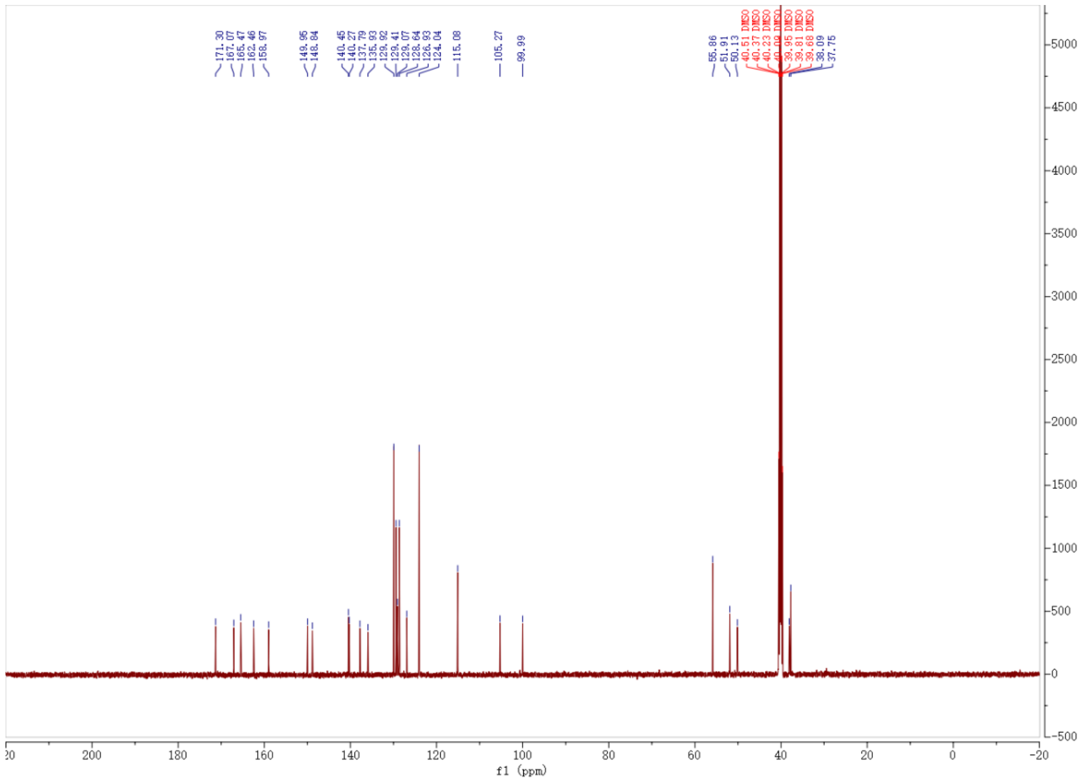

171.30
167.07
165.47
162.46
158.97
149.95
148.84
140.45
140.27
137.79
135.93
129.92
129.41
129.07
128.64
126.93
124.04
115.08
105.27
99.99
55.86
51.91
50.13
40.51 DMSO
40.37 DMSO
40.23 DMSO
40.09 DMSO
39.95 DMSO
39.81 DMSO
39.68 DMSO
38.09
37.75
f1 (ppm)

1.15 MS, ^{1}H-NMR and ^{13}C-NMR Spectra for TD-1m

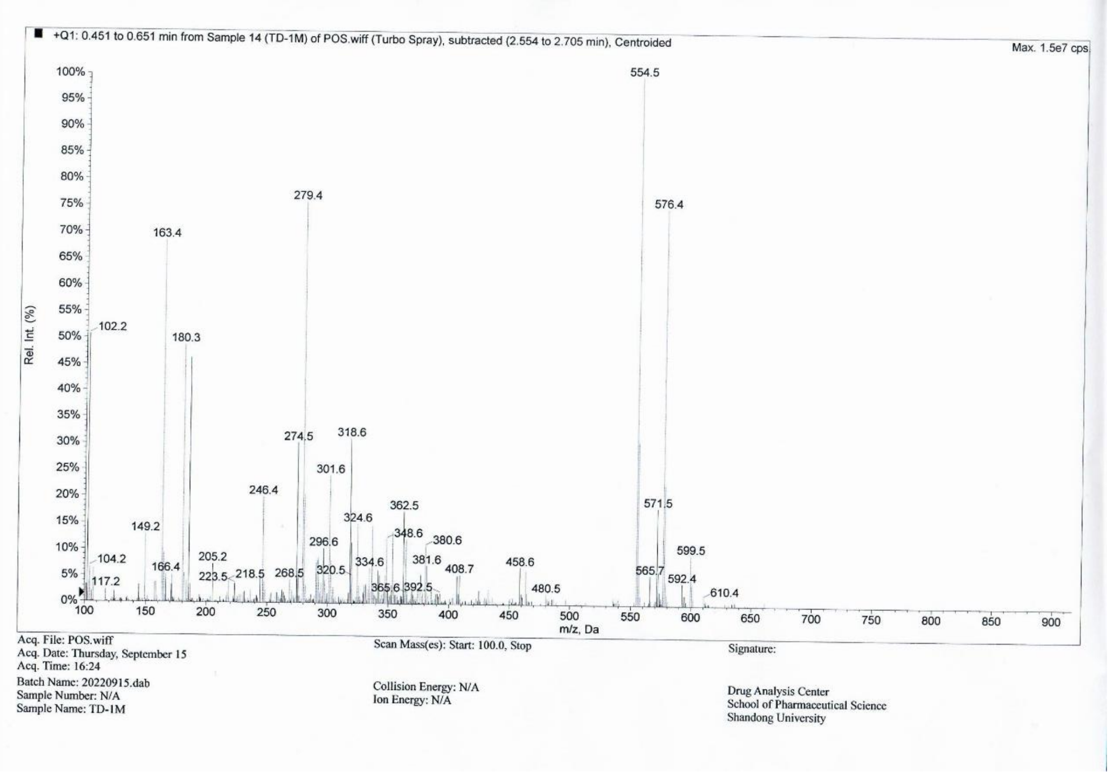

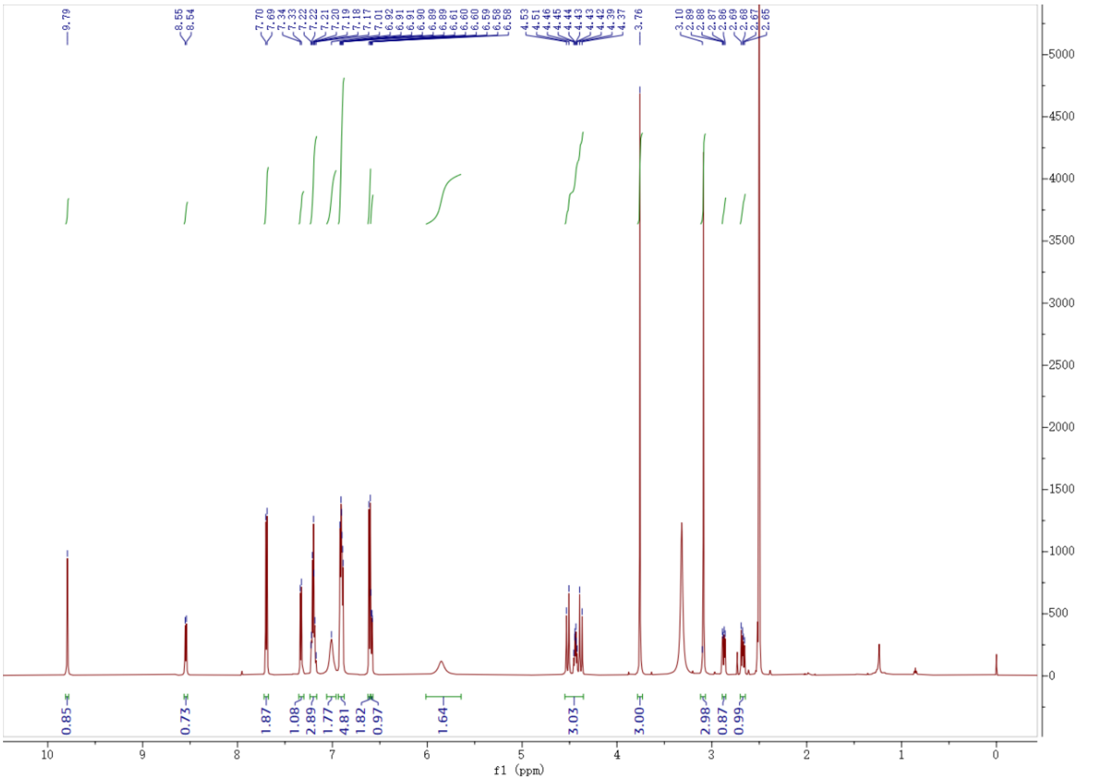

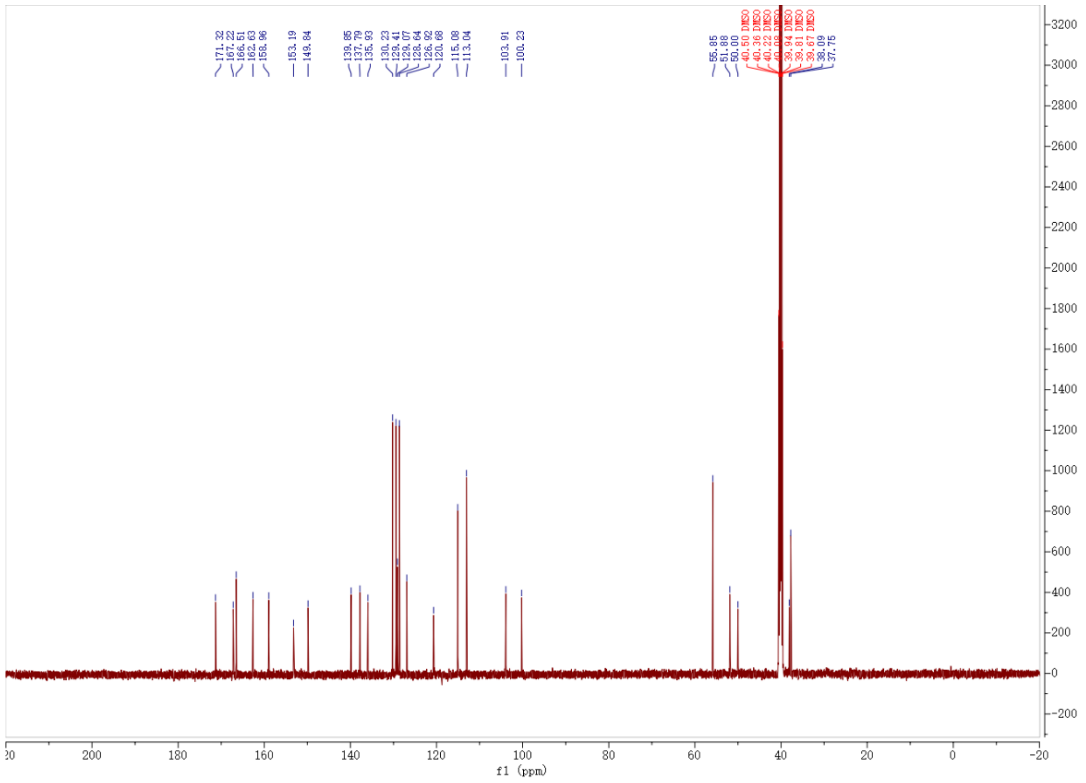

171.32
167.22
166.51
162.63
158.96
153.19
149.84
139.85
137.79
135.93
130.23
129.41
129.07
128.64
126.92
120.68
115.08
113.04
103.91
100.23
55.85
51.88
50.00
40.50 DMSO
40.36 DMSO
40.22 DMSO
40.08 DMSO
39.94 DMSO
39.81 DMSO
39.67 DMSO
38.09
37.75
f1 (ppm)

2 Standard Curve of Water Solubility

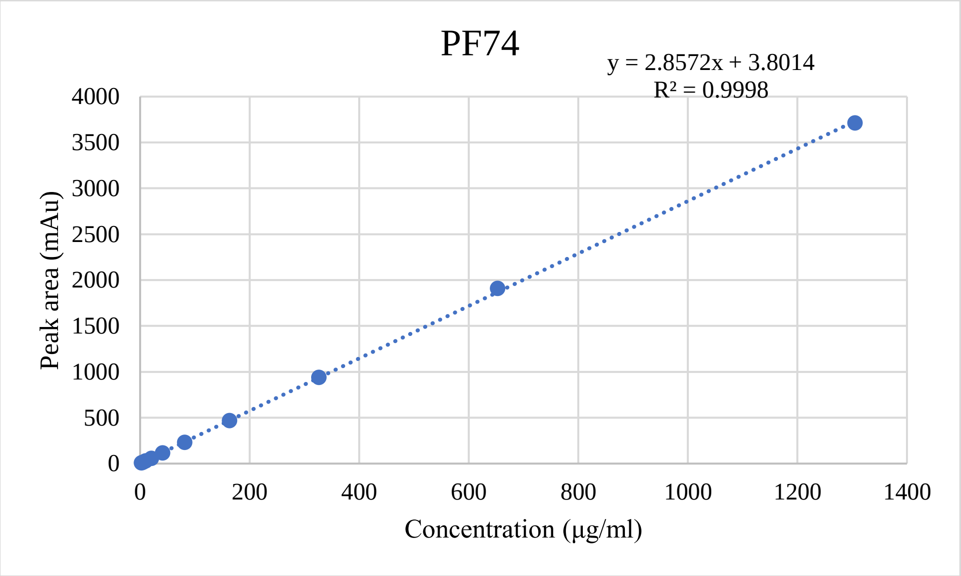

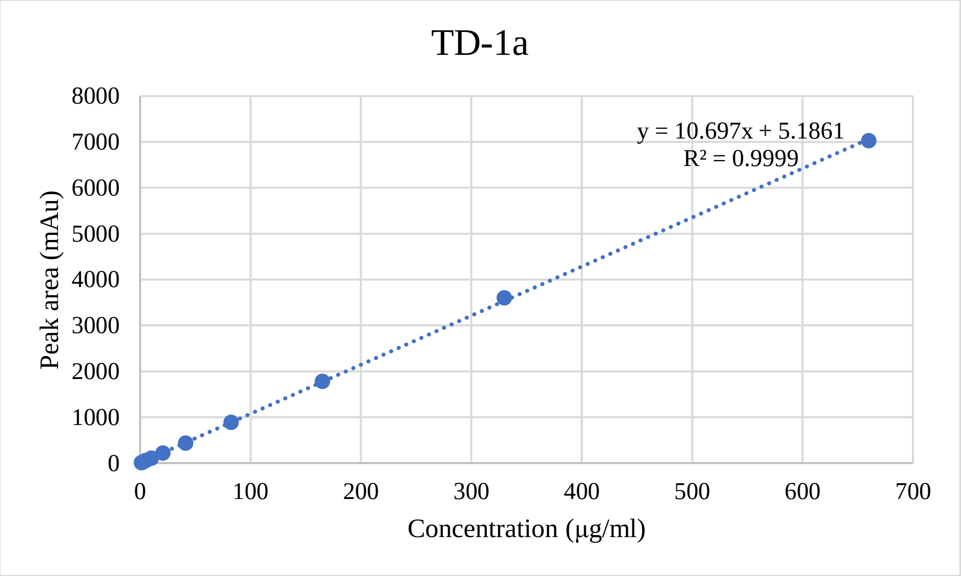

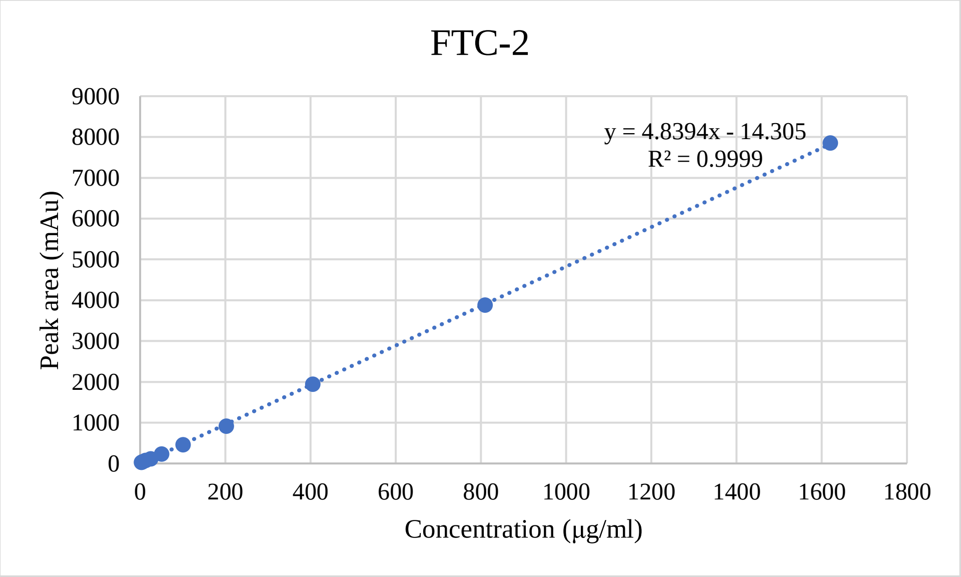

Supplement: Supplementary file 1 [file molecules-27-07640-s001.zip › molecules-1963643-supplementary.pdf]
